# Neuregulin 1 Drives Morphological and Phenotypical Changes in C2C12 Myotubes: Towards *De Novo* Formation of Intrafusal Fibres *In Vitro*

**DOI:** 10.3389/fcell.2021.760260

**Published:** 2022-01-11

**Authors:** Philip Barrett, Tom J. Quick, Vivek Mudera, Darren J. Player

**Affiliations:** ^1^ Centre for 3D Models of Health and Disease, Division of Surgery and Interventional Science, Faculty of Medical Sciences, University College London, London, United Kingdom; ^2^ Peripheral Nerve Injury Research Unit, Royal National Orthopaedic Hospital, London, United Kingdom; ^3^ UCL Centre for Nerve Engineering, University College London, London, United Kingdom

**Keywords:** muscle spindle, intrafusal, proprioception, neuromuscular, mechanoreceptor, afferents, skeletal muscle, myotubes

## Abstract

Muscle spindles are sensory organs that detect and mediate both static and dynamic muscle stretch and monitor muscle position, through a specialised cell population, termed intrafusal fibres. It is these fibres that provide a key contribution to proprioception and muscle spindle dysfunction is associated with multiple neuromuscular diseases, aging and nerve injuries. To date, there are few publications focussed on *de novo* generation and characterisation of intrafusal muscle fibres *in vitro.* To this end, current models of skeletal muscle focus on extrafusal fibres and lack an appreciation for the afferent functions of the muscle spindle. The goal of this study was to produce and define intrafusal bag and chain myotubes from differentiated C2C12 myoblasts, utilising the addition of the developmentally associated protein, Neuregulin 1 (Nrg-1). Intrafusal bag myotubes have a fusiform shape and were assigned using statistical morphological parameters. The model was further validated using immunofluorescent microscopy and western blot analysis, directed against an extensive list of putative intrafusal specific markers, as identified *in vivo*. The addition of Nrg-1 treatment resulted in a 5-fold increase in intrafusal bag myotubes (as assessed by morphology) and increased protein and gene expression of the intrafusal specific transcription factor, Egr3. Surprisingly, Nrg-1 treated myotubes had significantly reduced gene and protein expression of many intrafusal specific markers and showed no specificity towards intrafusal bag morphology. Another novel finding highlights a proliferative effect for Nrg-1 during the serum starvation-initiated differentiation phase, leading to increased nuclei counts, paired with less myotube area per myonuclei. Therefore, despite no clear collective evidence for specific intrafusal development, Nrg-1 treated myotubes share two inherent characteristics of intrafusal fibres, which contain increased satellite cell numbers and smaller myonuclear domains compared with their extrafusal neighbours. This research represents a minimalistic, monocellular C2C12 model for progression towards *de novo* intrafusal skeletal muscle generation, with the most extensive characterisation to date. Integration of intrafusal myotubes, characteristic of native, *in vivo* intrafusal skeletal muscle into future biomimetic tissue engineered models could provide platforms for developmental or disease state studies, pre-clinical screening, or clinical applications.

## Introduction

Muscle spindles (MS) are mechanosensory organs that detect and mediate static and dynamic information about skeletal muscle fibre length, as well as rate and extent of strain (Thornell et al., 2015). There are three sub-types of intrafusal fibre; nuclear bag_1_, bag_2_ and chain, each have a unique morphology, innervation pattern and protein expression profiles, which contribute to a distinctive functional response to static and dynamic muscle stretch. Nuclear bag fibres have a distinctive fusiform shape with a clustering of nuclei at the equatorial region, nuclear chain fibres have a linear morphology and nuclear alignment (Macefield and Knellwolf, 2018). MS provide a significant contribution to proprioceptive function, which can be described as the sense of position and movement of parts of the body relative to one another (Macefield and Knellwolf, 2018). Impairment of proprioception and dysfunction of the MS is linked with many neuromuscular diseases including; multiple sclerosis (Feys et al., 2011; Prather et al., 2011; Fling et al., 2014) Parkinson’s disease (Conte et al., 2013; Teasdale et al., 2017), muscular dystrophy (Ovalle and Dow, 1986), and peripheral nerve injuries (Cope et al., 1994; Haftel, 2005; Ruijs et al., 2005; Maas et al., 2007; Bullinger et al., 2011; Prather et al., 2011). Proprioceptive function also deteriorates in diabetic patients (van Deursen et al., 1998; Muller et al., 2008; Muramatsu et al., 2017) and during aging (Miwa et al., 1995; Shaffer and Harrison, 2007). Proprioceptive dysfunction can cause a considerable alteration in the regulation of the speed and precision of limb movement. These effects cause significant physical limitations, including; disruption to balance, locomotion and postural stability (van Deursen et al., 1998; van Deursen and Simoneau, 1999; D’Silva et al., 2016; Ettinger et al., 2018; Ferlinc et al., 2019), which can significantly negatively impact upon the individual’s quality of life (Teasdale et al., 2017).

The muscle spindle proprioceptive system have been extensively studied *in vivo*, whereby studies conducted in mice, rats and cats have formed the basis of much of our current understanding (Banks, 1994, 2015; Macefield and Knellwolf, 2018). Intrafusal fibres develop from primary myotubes, whereby the onset of intrafusal specification coincides with Ia afferent innervation and initiates spindle morphogenesis (Chal and Pourquié, 2017). Approaching Ia afferents release neuregulin-1 (Nrg-1), which binds primary muscle tyrosine kinase receptors ErbB (2-4) (erythroblastic leukemia viral oncogene homologue), resulting in downstream expression of the early growth response protein-3 (Egr3) (Tourtellotte and Milbrandt, 1998; Tourtellotte et al., 2001; Albert et al., 2005; Oliveira Fernandes and Tourtellotte, 2015). Disrupting any of these key mechanisms *in vivo* results in aberrant MS development and function (Tourtellotte and Milbrandt, 1998; Andrechek et al., 2002; Hippenmeyer et al., 2002; Leu et al., 2003; Jacobson et al., 2004; Albert et al., 2005; Herndon et al., 2014; Oliveira Fernandes and Tourtellotte, 2015). To date most skeletal muscle models often neglect the integrated nature of the neuromuscular system by not accounting for afferent functions mediated via the muscle spindle. Such experiments are expensive, time consuming and extremely difficult *in vivo* considering the an adult human contains only about 50,000 muscle spindles (Kröger and Watkins, 2021).

Therefore, *in vitro* models of the MS offer the possibility to study the cellular and molecular mechanisms regulating development, function and the effect of injury and disease, in a highly controlled environment. To develop a representative model *in vitro*, it is necessary to identify and recapitulate native tissue characteristics. *In vivo*, postnatal intrafusal fibres contain a higher number of paired-domain transcription factor 7 (PAX7) positive satellite cells and retain the expression of embryonic satellite cell marker, paired-domain transcription factor 3 (PAX3) (Horst et al., 2006; Kirkpatrick et al., 2008, 2010). Their myonuclei maintain the expression of an early myogenic regulatory factor (MRF), Myf5 (Zammit et al., 2004), they remain comparatively small and have reduced myonuclear domains (volume of sarcoplasm per myonucleus) compared to extrafusal fibres (Kozeka and Ontell, 1981). They also have preferential expression of embryonic (MyHC3), neonatal (MyHC8) and specialised (MyHC6, MyHC7b) myosin heavy chains (MyHC) (Walro and Kucera, 1999; Liu et al., 2002), concomitant with the retained expression of developmental protein, Egr3 (Tourtellotte and Milbrandt, 1998; Oliveira Fernandes and Tourtellotte, 2015). To this end, when developing an *in vitro* model, it would be prudent to measure the characteristics outlined above.

Despite this, characterisation *in vitro* has largely relied on morphological identification of what have been termed “bag myotubes” (intrafusal nuclear bag fibres), alongside expression of developmental proteins or putative phenotypical proteins of intrafusal fibres as characterised *in vivo* (Jacobson et al., 2004; Rumsey et al., 2008; Colón et al., 2017, 2020; Guo et al., 2017; Qiao et al., 2018)*.* Previous *in vitro* studies for the purpose of developing *de novo* intrafusal muscle fibres have indicated that Nrg-1 treatment (100–160 ng/ml, 12.5–20 nM) causes an increase in bag-like myotubes with an expanded equatorial region and centrally clustered nuclei in primary human and rat cells (Rumsey et al., 2008; Colón et al., 2017; Guo et al., 2017; Qiao et al., 2018), paired with high magnification images of bag myotube specific MyHC6 and Egr3 staining. (Rumsey et al., 2008; Colón et al., 2017). To date, the only quantitative protein (western blot) analysis of myogenic cells following Nrg-1 (8 ng/ml, 1 nM) treatment indicated a sharp increase of approximately 6-fold in both MyHC8 and a slow developmental isoform (Jacobson et al., 2004) (in other papers this refers to the s46 antibody (Tourtellotte et al., 2001; Albert et al., 2005), which recognises MyHC6) (Jacobson et al., 2004). However, unlike the Hickman papers, MyHC6 immunoreactivity is not exclusive to the Nrg-1 treated myotubes and there is no mention of gross morphological changes following Nrg-1 treatment and therefore no mention of bag myotube specificity associated to MyHC6.

Current models have now progressed rapidly to incorporate afferent and efferent innervation with induced pluripotent stem cell (iPSC)-derived intrafusal myotubes, while overlooking some basic structural and molecular characterisation (Colón et al., 2017; Guo et al., 2017). The extent of *de novo*, *in vitro* intrafusal fibres structural and physiological similarity to native intrafusal fibres is not yet fully elucidated. Therefore, there is a need to further characterise cell engineered, Nrg-1 induced intrafusal myotubes before they can become platforms to integrate muscle spindle associated proprioceptive physiology, disease state modelling or clinical applications. Furthermore, despite the advances in iPSC and primary cell technologies, there are significant challenges with using these cells. Myogenic cell lines (e.g., the C2C12 cell line) provide established, fusion-competent cells, are affordable, sustainable and are easy to maintain. However, the capacity for intrafusal myotube differentiation following Nrg-1 supplementation in myogenic cell lines is currently unknown.

Current literature relies a subjective determination of intrafusal morphology by the investigator (Rumsey et al., 2008, 2010; Colón et al., 2017, 2020; Guo et al., 2017). To improve objectivity, statistical morphological parameters for assigning intrafusal bag myotubes from a heterogeneous population need to be defined. Secondly, MyHC isoform protein sequences are very similar, which has suggested limitations caused by cross-reactivity of antibodies (Thornell et al., 2015). Since MyHC expression is controlled at the transcriptional level (Schiaffino et al., 2015), MyHC gene expression could be a suitable alternative to detect intrafusal specific patterns (Brown et al., 2012).

In addition, there are muscle spindle specific targets*,* which have not yet been applied for *in vitro* characterisation. These are; Ets variant 4 (Etv4) (Arber et al., 2000; Hippenmeyer et al., 2002), Glial cell derived neurotrophic factor (Gdnf) (Shneider et al., 2009; Schiaffino and Reggiani, 2011), neurotrophin-3 (NT3) (Chen et al., 2002; Kröger and Watkins, 2021), low- affinity neurotrophin receptor p75 (Ngfr), somatostatin receptor type 2 (Sstr2) and the type III inter- mediate filament peripherin 1 (Prph1) (Albert et al., 2005).

*In vitro* skeletal muscle research has gained momentum over the past 10 years, however there are still relatively few publications tackling cell and tissue engineering approaches to generating *de novo* intrafusal muscle fibres *in vitro* (Barrett et al., 2020) and they are not without some inconsistencies, described previously. This study presents an objective, novel method for assignment of intrafusal bag morphology and the most in-depth characterisation of myotubes following Nrg-1 treatment to-date. The aim was to determine the degree to which Nrg-1 induces an intrafusal fibre-like morphology and the extent the myotubes recapitulate features of native intrafusal fibres. Developing a well characterised *in vitro* model of intrafusal fibres using C2C12 myoblasts, will provide an investigative tool for developmental physiology, the framework for multi-cell, innervated, integrated models of the muscle spindle and relevant disease models in a defined, controlled and highly reproducible manner.

## Methods

### Cell Culture

**FIGURE 1:**
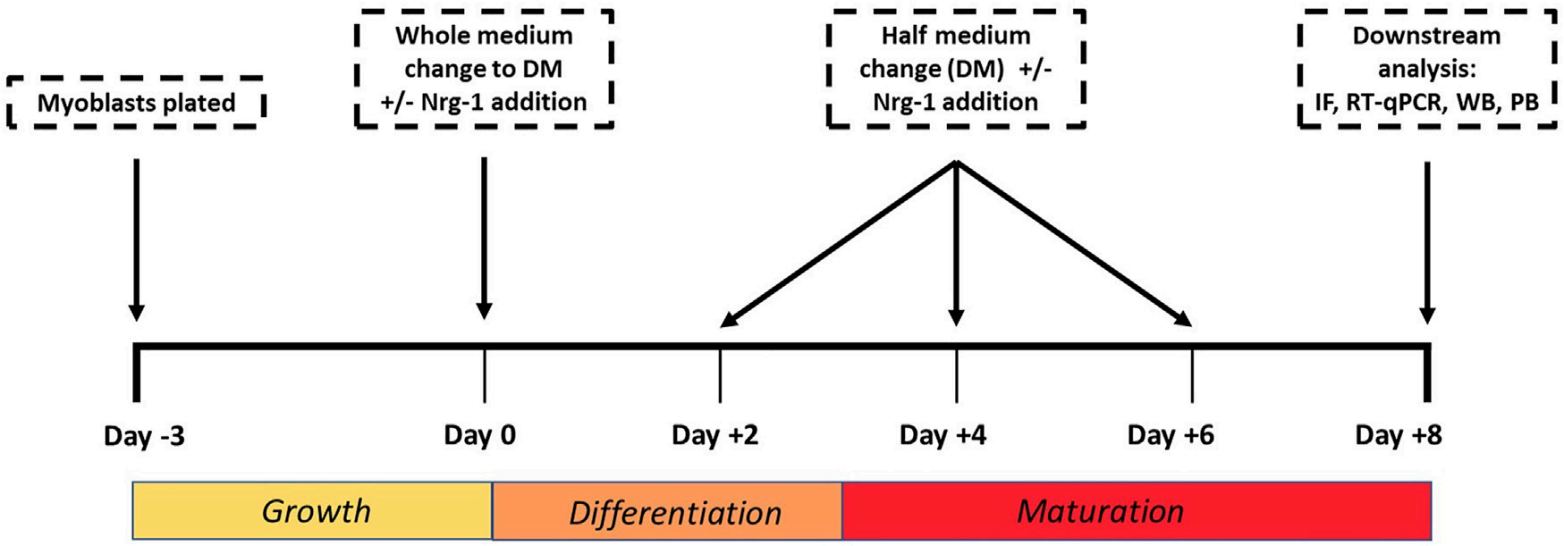
Cell culture experimental timeline used to tissue engineer intrafusal skeletal muscle fibres for downstream analysis. IF (Immunofluorescence microscopy), RT- qPCR (Reverse Transcription Quantitative Polymerase Chain Reaction), WB (Western Blot), PB (PrestoBlue).

C2C12 cells were seeded at a density of 10,000 cells/cm^2^ in growth medium (GM), consisting of high glucose Dulbecco’s modified eagle’s medium (DMEM; Sigma-Aldrich, United Kingdom), 10% foetal bovine serum (FBS; Gibco, United Kingdom), 1% antibiotic/antimycotic solution (AS; HyClone^TM^ from Thermo Fisher, United Kingdom) until confluent (3 days) at 37°C and 5% CO_2_. At day 0, cells were encouraged to differentiate by replacing the medium to low serum differentiation medium (DM) consisting of DMEM, 2% Horse serum (HyClone^TM^ from Thermo Fisher, United Kingdom) and 1% AS and cultured for 8 days at 37°C and 5% CO_2_ (Figure 1). Half DM was replaced every 48 h. To induce Intrafusal fibre differentiation, DM was supplemented with 100 ng/ml recombinant Nrg-1 (R&D systems, United States).

### Immunofluorescent Microscopy

Adherent cells in 24 well plates were fixed with 4% paraformaldehyde diluted in molecular grade water for 15 min at room temperature. Cells were permeabilized with 0.25% Triton X-100 in (Sigma-Aldrich, United Kingdom) PBS and blocked with 5% Horse serum (HyClone^TM^ from Thermo Fisher, United Kingdom) for 30 min. Fixed and permeabilized cells were incubated with primary antibodies Egr3 (Santa Cruz Biotechnology, United States, c-390967), MyHC3 (Santa Cruz Biotechnology, sc-53091), MyHC6 (Novus Biologicals, United States, NB300-284) or MyHC8 (Invitrogen™, PA5-72846 through Thermo Fisher Scientific, United Kingdom) at 1:200 at 1:100, 1:100, 1:300 and 1:200, respectively, then incubated at 37°C for 2 h. Secondary antibodies Goat Anti-Mouse 488 (Abcam, United Kingdom, ab150113) or Goat Anti-Rabbit (Abcam, United Kingdom, ab150077) were incubated at 1:1,000 concurrently with NucBlue^TM^ DAPI (Thermo Fisher, United Kingdom) and Phalloidin (Alexa Fluor, A12380) for 1 h at room temperature. Prior to imaging, PBS was removed from the wells and cells were imaged using a ZEISS Axio Observer or ZEISS LSM 880 fluorescent microscope.

### Egr3 Staining Intensity Calculations

All images were taken using the same camera and settings on Zeiss Axio Observer. In Fiji (Schindelin et al., 2012), background fluorescence was removed from images prior to experimental intensity measurements. The background fluorescence was determined by a threshold that removes 99% of positive pixel coverage (grey scale intensity above 0) from a primary negative image. Egr3 image coverage was determined as the total pixels expressing above background fluorescence divided by the total image pixels. Egr3 intensity per nuclei was calculated by dividing total image pixel intensity above background by total image nuclei number. Considering the majority of Egr3 expression occurs in the nucleus, and nuclei numbers are different between groups, making Egr3 expression relative to nuclei number negates the effect of nuclei number on Egr3 expression. Egr3 positive nuclei were visualised following background subtraction by comparing overlap with the DAPI channel.

### Quantification of Myotube Morphogenic Parameters

**FIGURE 2:**
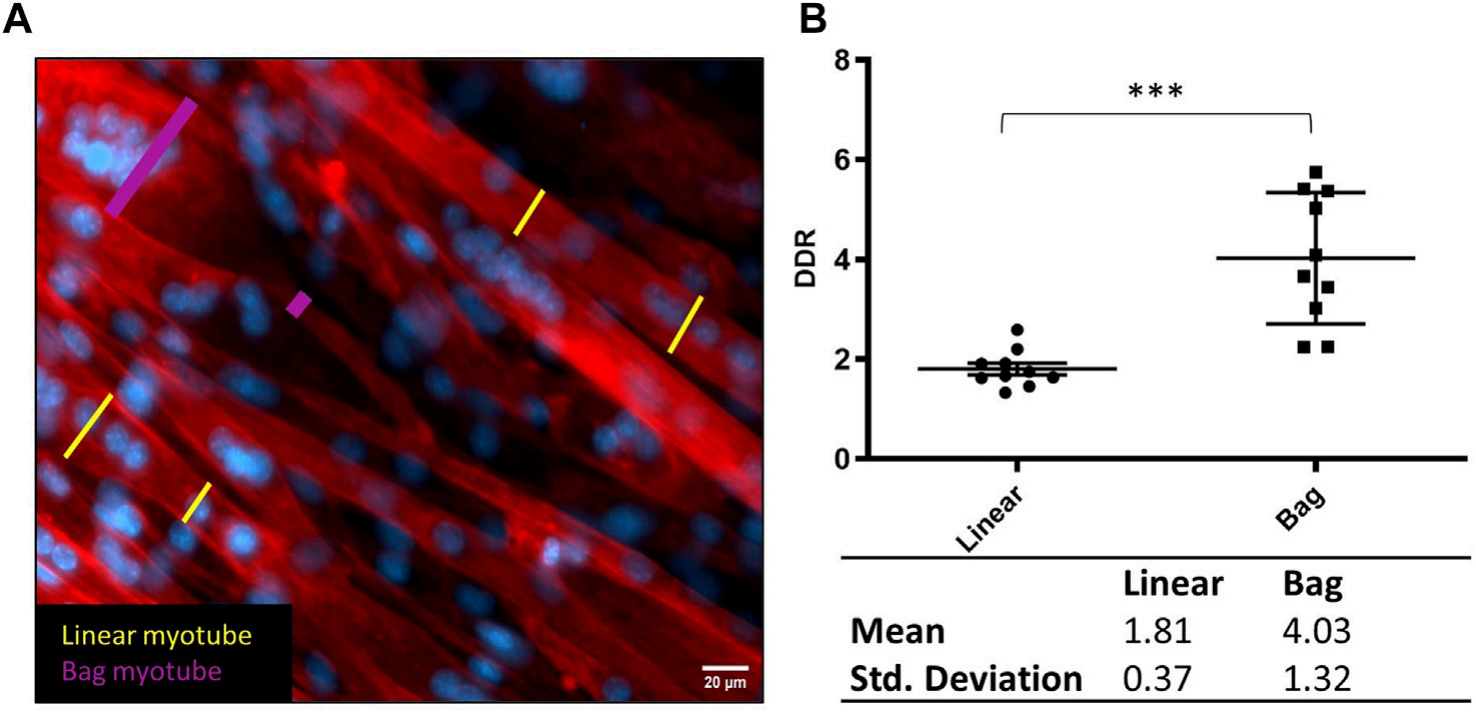
Defining bag and linear myotubes using Diameter Difference Ratio (DDR). **(A)** Representative fluorescent image with diameter measurements from linear myotubes in yellow and bag myotubes in pink. Phalloidin (red) DAPI (blue) (scale bar, 20 µm). **(B)** Scatter dot plot comparing DDR in Linear and Bag myotubes. Table below displays the mean and standard deviation from the graph which were used to determine the range for myotube populations. Mean ± SEM, *n* = 10, ***p < 0.001.

Using fluorescent micrographs from the control cultures at 8 days differentiation, 10 myotubes were manually identified from the mix of images from three biological repeats, each with three technical repeats. They were assigned as either having a typical linear (uniform shape and linear nuclei arrangement), assigned as linear myotube or bag-like morphology (bulging equatorial region with clustered nuclei (Banks, 2015), assigned as bag myotube. A myotube is defined by a single fibre, as defined by actin stain (phalloidin) and containing three or more nuclei (DAPI). The largest and smallest diameter, clearly visible and not interfered by an overlapping myotube were measured in Fiji and used to calculate a diameter difference ratio (DDR) between those measures. Plotting DDR defines two distinct, statistically significantly populations for linear and bag myotubes (Figure 2). The Mean DDR ratio of linear and bag fibres was 1.81 ± 0.37 and 4.03 ± 1.32, respectively (*p* < 0.001). The standard deviation sets the range of the two distinct myotube populations: Linear myotube ≤ 2.18, Bag myotube ≥ 2.71, 2.18 < Unassigned myotube < 2.71. This novel method of identifying intrafusal bag fibre morphologies from a heterogeneous myotube population eliminates a significant degree of investigator subjectivity and bias. Fusion efficiency was calculated as the total number of nuclei within myotubes divided by the total image nuclei.

### RNA Extraction, cDNA Synthesis and RT-qPCR

RNA was extracted using the phase separation TRI Reagent^®^ and chloroform method (Rio et al., 2010) (reagents from Sigma-Aldrich, United Kingdom). The total RNA obtained was quantified and tested for integrity using the NanoDrop™. The RNA was then transcribed into cDNA using the High-Capacity cDNA Reverse Transcription Kit (Applied Biosystems through Fisher Scientific, United Kingdom) on the T100™ Thermal Cycler (Bio-Rad, United Kingdom). Primers were designed using Primer-BLAST (Ye et al., 2012) for an annealing temperature (Ta) of 60°C, with exception of Myh4 (Zhou et al., 2010), Myh6 (Zhou et al., 2010), Csnk2a2 (Hildyard and Wells, 2014) and Myf5 (Brown et al., 2012). Primer sequences are shown in Table 1 and were ordered from Sigma-Aldrich (United Kingdom). To be included in the study, primers needed an efficiency of 90–115%, an *r* ^2^ over 0.95 and contain a single melting peak (Bustin et al., 2009). Myh15 is missing due to very low CT expression at the experimental cDNA concentration ranges. Gene target amplification utilised the iTaq™ Universal SYBR^®^ Green Supermix on the CFX96™ Touch System (Bio-Rad, United Kingdom). Reactions wells were 10 μl, containing 20 ng of sample cDNA, alongside a primer concentration of 0.2 μM. Reactions were carried out in triplicate on 96 well plates. Samples were pre-incubated at 95°C for 5 min followed by 40 PCR amplification cycles (denaturation: 95°C for 10°s; annealing & extension: 60°C 30°s), followed by a melt curve analysis. MIQE guidelines were followed to ensure experimental transparency and repeatability (Bustin et al., 2009). Relative gene expression was calculated using the ΔCt and 2-ΔΔCt method (Schmittgen and Livak, 2008), normalising to the reference gene casein kinase 2, alpha prime polypeptide (Csnk2a2) and expression made relative to the experimental control mean for each plate. To compare the percentage composition of Myh (Figure 4B), Target Myh expression was made relative to total Myh delta CT expression for that individual sample. When repeated for all detectable Myh genes, data can be used to display the proportional representation of Myh gene expression.

**TABLE 1:** Sequence, accession code and efficiency of primers used for mRNA analysis.

Gene	Primer sequence 5′–3′		Efficiency (%)	Accession code
	Forward	Reverse		
*Myh1*	GGC​CTA​CAA​GAG​ACA​AGC​TGA	ACT​TTC​CTG​CAC​TTG​GAT​CA	102.3	NM_030679.2
*Myh2*	TCT​AAG​GCC​AAG​GGA​AAC​CTC	TAC​CAG​CGC​TTC​CTT​CTC​ATC	107.3	NM_001039545.2
*Myh3*	TCG​CTA​CAA​CAG​ATG​CGG​AC	CCT​GGG​GTC​TTG​GTT​TCG​TT	92.4	NM_001099635.1
*Myh4*	CAC​CTG​GAC​GAT​GCT​CTC​AGA	GCT​CTT​GCT​CGG​CCA​CTC​T	102.4	NM_010855.3
*Myh6*	CCA​ACA​CCA​ACC​TGT​CCA​AGT	AGA​GGT​TAT​TCC​TCG​TCG​TGC​AT	101.3	NM_001164171.1
*Myh7*	TAC​TTG​CTA​CCC​TCA​GGT​GGC​T	TGT​CAT​CGG​GCA​CAA​AAA​CAT​C	90.2	NM_080728.3
*Myh8*	ACG​CTA​GTG​CTG​AAG​CAG​ATG​G	ACC​GTA​CGA​AGT​GAG​GGT​GT	103.7	NM_177369.3
*Myh13*	CGG​CAA​GAA​GCA​GAT​CCA​GA	TCC​TCG​GCC​TGG​TAA​GTC​A	97.4	NM_001081250.2
*Myh7b/14*	AGA​CCA​GAA​GGT​GCT​GAC​AGT	CCG​GGA​GCC​ATT​TGT​ATG​GG	106.6	NM_001085378.2
*Csnk2a2*	CAC​CAA​CAA​TGA​GAG​GGT​GG	GGT​GTC​TTT​GAC​ACA​GGG​TCC	97.9	NM_009974.3
*Myf5*	TGA​CGG​CAT​GCC​TGA​ATG​TA	GCT​CGG​ATG​GCT​CTG​TAG​AC	96.0	NM_008656.5
*Myod1*	TGC​TCT​GAT​GGC​ATG​ATG​GAT​T	AGA​TGC​GCT​CCA​CTA​TGC​TG	98.1	NM_010266.2
*Myog*	ATC​CAG​TAC​ATT​GAG​CGC​CT	CAA​ATG​ATC​TCC​TGG​GTT​GGG	100.2	NM_031189.2
*Egr3*	CCG​GTG​ACC​ATG​AGC​AGT​TT	TTG​GGC​TTC​TCG​TTG​GTC​AG	106.5	NM_018781.4
*Gdnf*	TGA​CCA​GTG​ACT​CCA​ATA​TG	GTT​TAT​CTG​GTG​ACC​TTT​TCA​G	111.7	NM_010275.3
*Prph*	AGC​TAC​TGG​AAG​GGG​AGG​AG	TCC​AGG​TCA​CTG​TGC​TGT​TC	92.0	NM_013639.2
*Sstr2*	GAG​AAC​ACA​GGG​AAG​CGA​GT	GCT​GCT​TTC​CAC​TCC​GTC​TA	110.5	NM_001042606.3
*Etv4*	CGA​GTG​CCC​TAC​ACC​TTC​TG	GGG​GAC​TTG​ATG​GCG​ATT​TG	92.9	NM_001316365.1

### Western Blotting

To lyse cells, RIPA buffer containing protease inhibitor at 1:100 dilution (both Sigma-Aldrich, United Kingdom) was used. Lysed cell suspensions underwent protein concentration quantification using the Pierce™ BCA Protein Assay Kit (Thermo Fisher, United Kingdom), as per the manufacturer’s instructions. Working solutions were made up to 0.8 μg/μl with RIPA and 2x Concentrate Laemmli Sample Buffer (Sigma-Aldrich, United Kingdom) and 20 μg per sample were loaded onto 4–15% Mini-PROTEAN^®^ TGX™ Precast 10-well protein gels and run at 200 V for approximately 40 min using the Mini-PROTEAN^®^ Tetra Cell and PowerPac™ 300 using tris-glycine SDS running buffer (all Bio-Rad, United Kingdom). Protein ladder SeeBlue™ Plus 2 Pre-stained Protein Standard (Invitrogen™ through Thermo Fisher, United Kingdom) was used. Proteins on the gels were then dry transferred onto nitrocellulose membranes using Trans-Blot^®^ Mini Nitrocellulose Transfer Packs and the Trans-Blot^®^ Turbo™ Transfer System (Bio-Rad, United Kingdom). Membranes were blocked for 1 h with 5% skimmed milk (Sigma-Aldrich, United Kingdom) in tris-buffered saline and 1% Tween 20 (TBST) (both Bio-Rad, United Kingdom), then incubated with 1° antibodies for Egr3, MyHC3, MyHC6 or MyHC8 (Same as immunofluorescent microscopy antibodies) at 1:250, 1:500, 1:500 and 1:500, respectively. Simultaneously incubating with loading control GAPDH at 1:10,000 (Abcam, United Kingdom, ab8245) in 5% skimmed milk overnight at 4°C followed by TBST washes. 2° antibodies IgG-HRP anti-mouse (Novus Biologicals, HAF018) and IgG-HRP anti-rabbit (Abcam, United Kingdom, ab6721) at 1:1,000 dilutions were incubated for 1 h in 3% skimmed milk, followed by three 15 min washes with TBST. Blots were then developed using Pierce™ ECL Western Blotting Substrate (Thermo Fisher, United Kingdom) and imaged using the ChemiDoc™ XRS imaging system and Image Lab™ software (Bio-Rad, United Kingdom). Quantification was performed in FIJI (Schindelin et al., 2012) and Microsoft Excel using an adjusted relative density method (Taylor et al., 2013).

### Cell Proliferation and Metabolic Assay

After the cell culture protocol explained above, DM plus 10% PrestoBlue^®^ (Thermo Fisher, United Kingdom) viability reagent was added to each well and incubated at 37°C for 2 h. Media was transferred in triplicates into 96 well plates and fluorescence read at 600–640 nm. Relative fluorescent unit (Rfu) readings were corrected to media only controls and then fold change from Nrg-1 treated cells was calculated relative to untreated controls. Cell counts were performed at Day 0 and Day +8 using NucBlue^TM^ Hoechst 33342 (Thermo Fisher, United Kingdom).

### Statistics

All statistical tests were performed in SPSS and GraphPad prism 9.0. *t*- test and ANOVA (with post-hoc Tukey test) analysis were completed in Graph pad 9.0. Firstly, the data was checked for normal distribution using Shapiro-Wilk test. If normally distributed, a student’s *t*-test or parametric ANOVA was used to ascertain significance. If not normally distributed, a Mann-Whitney *t* test was used. Egr3 intensity data was square root transformed prior to ANOVA analysis, providing a normally distributed data set (Bland and Altman, 1996). Nuclei Count data per myotube were analysed (treatment and fibre type effect) using negative binominal regression analysis, which accounted for data overdispersion and best fit the distribution of data (Hardin and Hilbe, 2014). All experiments, apart from western blot, were completed with an n of 9, consisting of three experimental (separate vial or plate) with three technical (well) repeats and one image from each repeat was analysed. Western Blot analysis had an *n* of 3, consisting of three experimental repeats ran (1 per lane) on three seperate blots. All raw data stated in the text is followed by ±Standard error of mean (SEM) when applicable.

## Results

### Nrg-1 Treated C2C12 Myotubes Contain Increased Intrafusal Bag Myotubes and Nuclei Number

**FIGURE 3:**
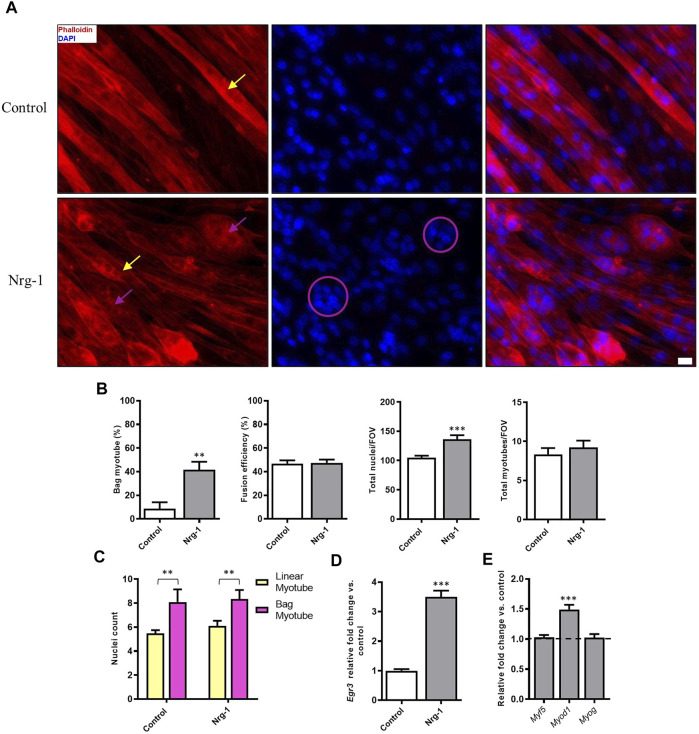
Nrg-1 treatment causes a significant increase in bag myotube formation and nuclei number in C2C12 myotubes. **(A)** High magnification representative fluorescent micrographs of cells ± Nrg-1 used for quantification. Phalloidin (red) and DAPI (blue), yellow arrows point toward myotubes with linear morphology and purple arrows toward bag morphology (Scale bar, 20 µm). **(B)** Comparison of key morphological criteria between myotubes ± Nrg-1. **(C)** Nuclei counts within bag and linear myotubes and ± Nrg-1. Control-Linear -60 myotubes, Control-Bag - 11 myotubes, Nrg-1-Linear - 40 myotubes, Nrg-1-Bag -33 myotubes. **(D)** qRT-PCR analysis of Egr3 expression. **(E)** qRT-PCR analysis of MRFs following Nrg-1 treatment vs. control. Control values are not shown and are represented by the dotted line at 1.0. Mean ± SEM, *n* = 9, **p < 0.01, ***p < 0.001.

To assess how Nrg-1 effects C2C12 myotube morphology, fluorescent microscopy targeting cell cytoskeleton (Phalloidin) and nuclei (DAPI) was used. Nrg-1 treatment leads a substantial change in myotube morphology (Figure 3A), in which there are visually more equatorially clustered nuclei with expanded myotube diameters. When analysed using DDR, this translates to an increase in bag myotubes from 8.52 ± 5.56% to 41.61 ± 6.77% (*p* < 0.01). Nuclei per field of view increased from 104.80 ± 3.56 in control to 136.30 ± 6.77 (*p* < 0.001) following Nrg-1 treatment (Figure 3B). There are no significant changes to other myogenic morphological criteria such as fusion efficiency (Control: 46.88 ± 2.68% v Nrg-1: 47.41 ± 2.82%) or myotubes per FOV (Control: 8.33 ± 0.82 v Nrg-1: 9.22 ± 0.88). In both control and treated populations, bag myotubes contain significantly more nuclei than linear fibres (8.09 ± 1.06 vs 5.48 ± 0.27 and 8.33 ± 0.76 vs 6.10 ± 0.43 respectively, both *p* < 0.01). The source of variation from myotube type was significant (*p* < 0.001) and insignificant for treatment (*p* > 0.05), meaning Nrg-1 treatment does not affect the average nuclei counts in each myotube type (Figure 3C). Relative Egr3 gene expression was quantified to confirm downstream Nrg-1 initiated Egr3 upregulation. Nrg-1 treated myotubes display a 3.5 ± 0.21-fold in Egr-3 gene expression (Figure 3D, *p* < 0.001), confirming sufficient downstream response. MRFs mediate myoblast proliferation and differentiation into myofibers (Zanou and Gailly, 2013) and provide an early indicator of myotube maturity. To this extent, MRF gene expression was quantified. The only significant difference was a 1.49 ± 0.08-fold increase in Myod1 (Figure 3F, *p* < 0.001). Together, the data from Figure 3 suggests that Nrg-1 upregulates Egr3, which correlates with a robust change of myotube morphology towards an intrafusal bag structure, without interfering with fusion efficiency or myotube numbers. In addition, bag myotubes contain significantly more nuclei than linear myotubes, contributing to increased nuclei counts following Nrg-1 treatment.

### Myosin Heavy Chain Gene Expression Significantly Altered in Nrg-1 Treated C2C12 Myotubes

**FIGURE 4:**
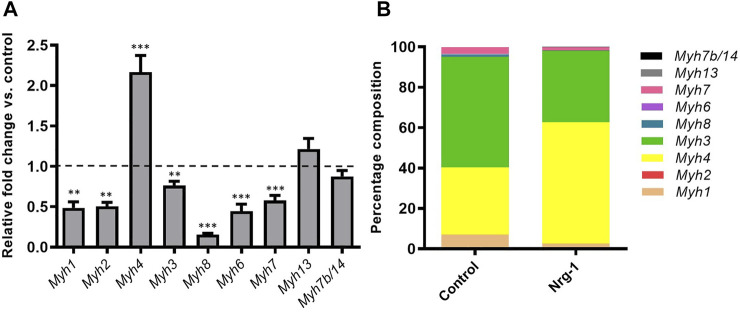
Nrg-1 treatment led to significant changes in Myh expression in all but two isoforms. **(A)** RT-qPCR analysis of all detectable Myh isoforms following Nrg-1 treatment vs control. Control values are not shown and are represented by the dotted line at 1.0. **(B)** Proportional representation for Myh expression. Mean ± SEM, *n* = 9, **p < 0.01, ***p < 0.001.

It is possible to identify intrafusal fibres *in vivo* based on the retained expression of immature (MyHC3 and MyHC8) and specialised (MyHC6 and MyHC7b/14) isoforms (Walro and Kucera, 1999; Liu et al., 2002). MyHC expression is regulated at a gene expression level (Schiaffino et al., 2015), therefore, MyHC gene (Myh) RT-qPCR analysis was completed to determine how Nrg-1 regulates Myh expression. All Myh isoforms, apart from Myh13 and Myh7b/14 displayed a significantly altered expression following Nrg-1 treatment (Figure 4A). Fold changes relative to control were as follows: Myh1, 0.49 ± 0.07 (*p* < 0.01), Myh2, 0.51 ± 0.05 (*p* < 0.01), Myh4, 2.17 ± 0.21 (*p* < 0.001), Myh3 0.76 ± 0.05 (*p* < 0.01), Myh8 0.16 ± 0.02 (*p* < 0.001), Myh6 0.44 ± 0.09 (*p* < 0.001) and Myh7, 0.58 ± 0.06 (*p* < 0.001).

Figure 4B uses the same data set as Figure 4A, however it is analysed and displayed as a relative proportional representation. This highlights the most biologically significant isoforms being represented. The majority of expression in both conditions comes from four genes: Myh1, Myh4, Myh3 and Myh7. Myh1 changes from 7.09 ± 0.87% to 2.68 ± 0.14%, Myh4 from 33.24 ± 0.85% to 60.13 ± 0.82%, Myh3 from 54.70 ± 1.72% to 35.28 ± 0.90% and Myh7 from 3.47 ± 0.22% to 1.63 ± 0.07%. This indicates the probable biological significance of Myh4 and Myh3 fold changes seen in Figure 4A, as they represent the greatest proportional expression in both control and Nrg-1 treated myotubes. This MyHC RT-qPCR data demonstrates a unique contractile phenotype in the treated population, however we see decreased expression in Myh isoforms associated with intrafusal fibres *in vivo* and *in vitro* (Barrett et al., 2020).

### Myosin Heavy Chain Protein Expression Significantly Altered in Nrg-1 Treated C2C12 Myotubes

**FIGURE 5:**
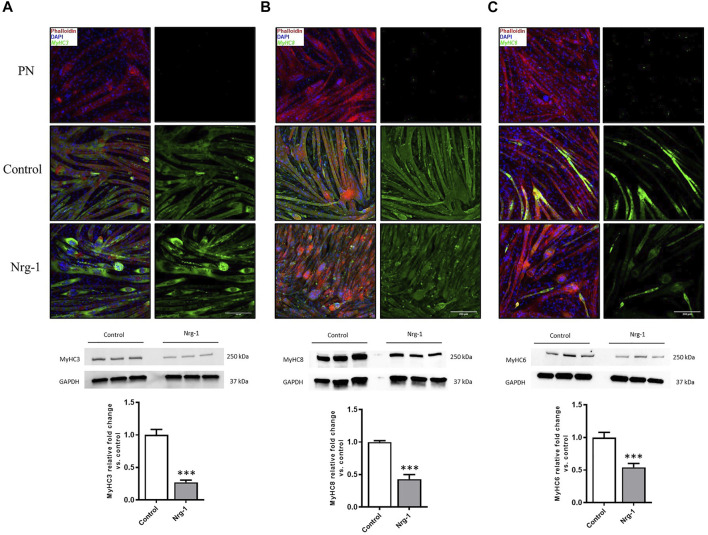
Decreased expression of putative intrafusal specific proteins following Nrg-1 treatment in C2C12 myotubes. Immunofluorescent imaging suggested no clear intrafusal bag myotube preferential expression MyHC3, 8 or 6. Phalloidin (red), DAPI (blue) and MyHC (green). Top to bottom: PN (primary negative), control and Nrg-1 treated representative fluorescent micrographs (scale bar, 200 µm), western blot representative gels and finally MyHC fold change relative to control based on western blots. **(A)** MyHC3. **(B)** MyHC8. **(C)** MyHC6. Western blot lanes represent three biological repeats, *n* of 3 achieved by repeating on three separate blots, Mean ± SEM, ***, *n* = 3, *p* < 0.001.

Despite MyHC expression being controlled at a transcriptional level (Schiaffino et al., 2015), analysis was conducted to establish whether gene expression changes resulted in consequential MyHC protein expression. To this extent, Immunofluorescence microscopy and western blot quantification was completed for MyHC3, MyHC8 and MyHC6. Immunofluorescence microscopy provides an opportunity to identify preferential expression (increased intensity) in myotubes with an intrafusal bag morphology compared to linear, providing a method to identify potential intrafusal bag myotube specific markers. Contradictory to previous literature, Nrg-1 treated myotubes did not display increased staining intensity for the aforementioned MyHCs, with MyHC6 actually appearing to have a lower staining intensity. Additionally, there was no obvious bag myotube specific staining (Figures 5A–C). Western blot analysis reveals a reduced expression across all tested MyHCs relative to control (Figures 5A–C): MyHC3 fold change relative to control was 0.27 ± 0.03, MyHC8 was 0.43 ± 0.07 and MyHC6 was 0.54 ± 0.06 (all *p* < 0.001). Gene expression changes from Figure 4 correlate with MyHC protein expression from Figure 5, highlighting reduced expression of putative intrafusal specific proteins and no bag myotube specific markers.

### Intrafusal Bag Myotubes Have an Increased Expression of Egr3 Protein Following Nrg-1 Treatment

At this point, results have presented a clear morphological change and an altered MyHC phenotype in C2C12 myotubes, following Nrg-1 treatment. However, there is no conclusive markers to define intrafusal fibres from extrafusal in this setup. Egr3 is a transcription factor essential for intrafusal myogenesis *in vivo* (Tourtellotte and Milbrandt, 1998; Tourtellotte et al., 2001; Andrechek et al., 2002; Hippenmeyer et al., 2002; Leu et al., 2003; Albert et al., 2005; Oliveira Fernandes and Tourtellotte, 2015) and has previously been associated with bag myotube morphology *in-vitro* (Rumsey et al., 2008, 2010; Colón et al., 2017, 2020; Guo et al., 2017). Therefore, Egr3 was visualised by immunofluorescence to ascertain whether its expression is dependent on treatment and/or morphology. Following this, western blots were performed for quantitative purposes. Finally, several gene candidates (identified from Albert, 2005) were quantified following Nrg-1 treatment to further characterise the extent of putative intrafusal-specific expression in this model.

**FIGURE 6:**
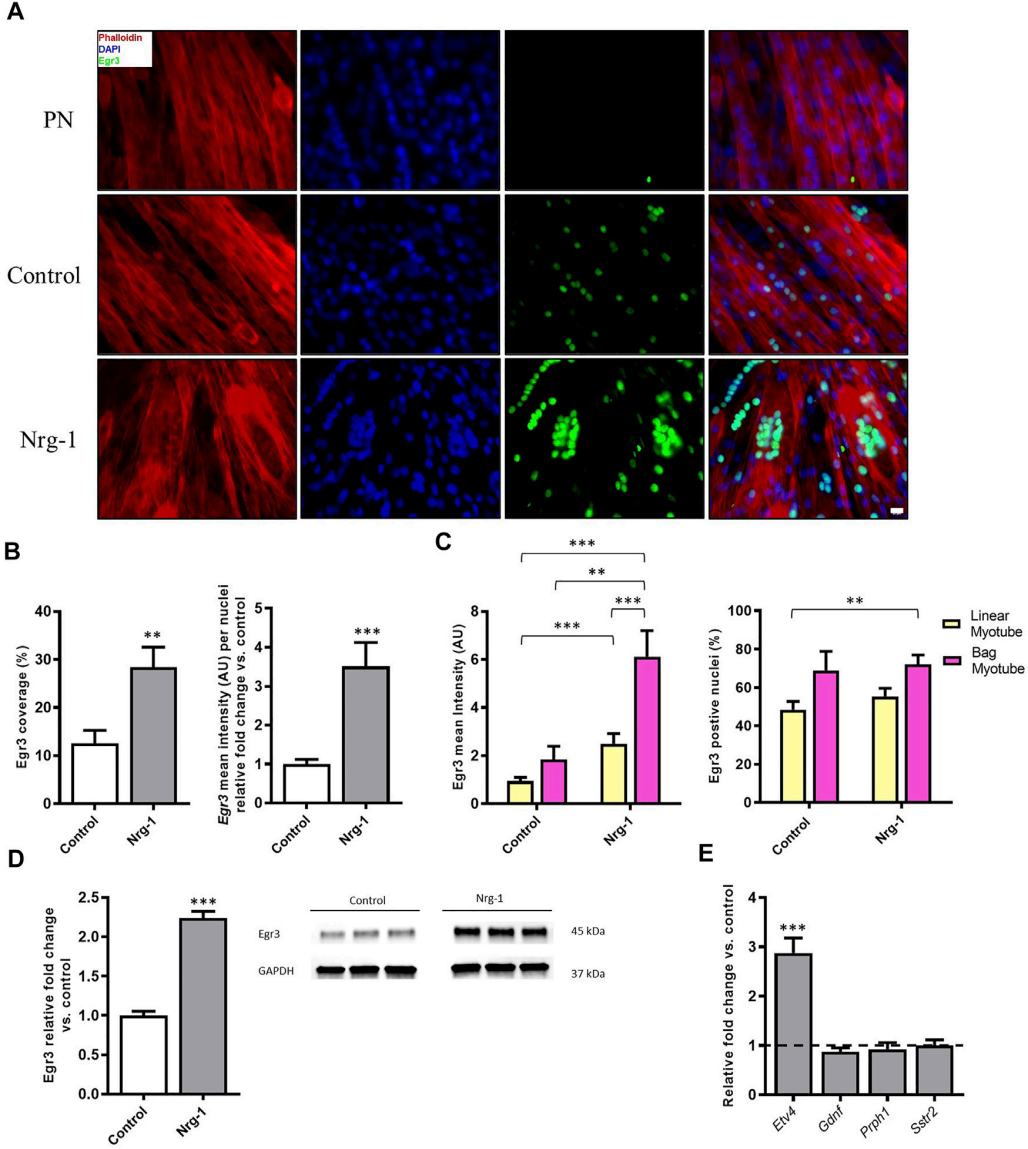
Increased expression of Egr3 protein in Nrg-1 treated C2C12 myotubes and intrafusal bag myotubes. **(A)** representative fluorescent micrographs following Nrg-1 treatment, (scale bar, 20 µm) Phalloidin (red), DAPI (blue) and Egr3 (green). **(B)** Percentage of Image covered by positive Egr3 expression (above threshold) and Egr3 intensity fold change per nuclei relative to control. **(C)** Two-way ANOVA analysis of treatment and myotube type, Left- Myotube Egr3 intensity Right- Egr3 positive nuclei %. Control Linear -52 myotubes, Control-Bag - 9 myotubes, Nrg-1-Linear - 47 myotubes, Nrg-1-Bag – 26 myotubes. **(D)** Western blot representative image and relative fold change for Egr3, each lane is a biological repeat (*n* = 3, from three blots). **(E)** Gene expression of additional putative intrafusal specific proteins relative to control. Mean ± SEM, *n* = 3, **p < 0.01, ***p < 0.001.

Egr3 image coverage (Control: 12.60 ± 2.66% vs Nrg-1: 28.37 ± 4.22%, *p* < 0.01) and staining intensity per nuclei (Control: 1.00 ± 0.12 vs Nrg-1: 3.51 ± 0.61, *p* < 0.001) increased following Nrg-1 treatment (Figure 6B). ANOVA analysis determined that myotube mean intensity of Egr3 was affected significantly by both treatment and myotube type (both *p* < 0.001) and yielded no interaction effect (*p* = 0.27). Tukey’s multiple comparisons post hoc test indicated significant differences between linear myotubes from control cells (control-linear, 0.88 ± 0.13) to linear myotubes from Nrg-1treated cells (Nrg-1-linear, 2.50 ± 0.42, *p* < 0.001) and bag myotubes from Nrg-1 treated cells (Nrg-1-bag, 6.13 ± 1.08, *p* < 0.001). There was a significant difference between Nrg-1-bag myotubes to bag myotubes from control cells (control-bag, 1.85 ± 0.54, *p* < 0.01) and Nrg-1-linear *p* < 0.001). Therefore, Egr3 staining intensity is increased in bag myotubes, and Nrg-1 treated myotubes (linear and bag), providing a possible marker for C2C12 derived intrafusal myotubes (Figure 6C).

The only significant source of variation in Egr3 positive nuclei percentage, was myotube type (*p* < 0.01), with a significant difference between control-linear (48.60 ± 4.60%) compared to Nrg-1-bag (72.12 ± 4.78%, *p* < 0.01). Indicating Nrg-1 treatment does not increase the percentage of nuclei expressing Egr3 (Figure 6C). In addition, in whole culture lysed population western blot data, Egr3 was increased 2.24 ± 0.08-fold (*p* < 0.001) following Nrg-1 treatment, corroborating with the increased expression visualised through immunofluorescence (Figure 6D). Finally, ETV4 mRNA expression was increased 2.88 ± −0.17-fold (*p* < 0.001) and Gdnf, Prph1 and Sstr2 mRNAs displayed no significant change (*p* > 0.05) (Figure 6E). Taken together, data from Figure 6 signifies that Nrg-1 treatment is upregulating Egr3 expression, particularly in myotubes with an intrafusal bag morphology (Figure 6C).

### Nrg-1 Treatment Causes Increased Cell Proliferation and Decreased Myotube Area per Myonuclei

**FIGURE 7:**
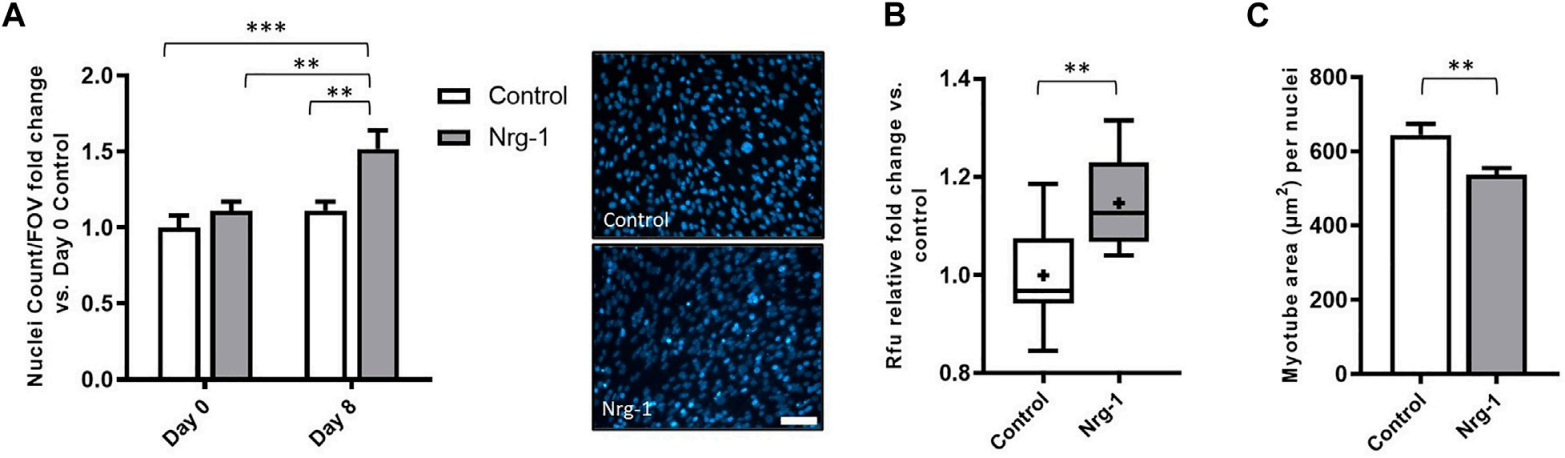
Nrg-1 treatment leads to cell proliferation during the differentiation phase in C2C12 cells. **(A)** Nuclei cells counts from day 0 and day 8 in both control and Nrg-1 treated myotubes relative to Day 0 control. To the right, representative fluorescent micrographs with Hoechst stain in blue (scale bar, 200 µm). **(B)** Rfu fold change relative to control from Prestoblue^TM^ cell viability assay. **(C)** Average myotube area (µm^2^) per myonuclei (using myotube data set from Figure 6). Mean ± SEM, *n* = 9, **p < 0.01, ***p < 0.001.

Figure 3C highlighted a significant increase in total nuclei/FOV, following Nrg-1 treatment after 8 days. To elucidate whether Nrg-1 was contributing to increased proliferation or reducing cell detachment and death, Hoechst nuclei stating was performed for both conditions at day 0 and day 8, followed by a Two-way ANOVA analysis. There was a significant source of variation from both treatment and time (*p* < 0.01), with no interaction effect. Tukey post-hoc comparisons identified the only significant increases between Day 8 Nrg-1 (1.52 ± 0.12) and all other conditions (Day 0 control: 1.00 ± 0.08, *p* < 0.001, Day 0 Nrg-1: 1.11 ± 0.06 and Day 8 Control: 1.112 ± 0.06, both *p* < 0.01), suggesting increased proliferation from day 0 to day 8 following Nrg-1 treatment (Figure 7A). To further clarify, Prestoblue^TM^ cell viability assay was performed, Nrg-1 treated myotubes displayed a relative fold increase of 1.15 ± 0.03 (*p* < 0.01), further suggesting Nrg-1 initiated proliferation (Figure 7B). Using the data set from Figure 6, the area of each myotube was divided by nuclei number to give a 2D *in vitro* version of myonuclear domain counts, which is the theoretical amount of sarcoplasm within a muscle fibre controlled by a single myonucleus (Teixeira and Duarte, 2011). Following Nrg-1 treatment, the average myotube area per nuclei reduced from 645.3 ± 30.08 µm to 537.9.0 ± 18.25 µm (*p* < 0.01) (Figure 7C). Nrg-1 is having a proliferative effect on C2C12s during the differentiation phase, resulting in increased nuclei counts and correlates with decreased myotube area per myonuclei.

## Discussion

There is an important clinical need to develop robust *in vitro* models of skeletal muscle that account for the sensory function of the muscle spindle through integration of intrafusal skeletal muscle fibres. This will help to investigate the basic molecular and cellular mechanisms regulating their phenotype in health and disease, in a controlled, defined, and ethical environment. In this study, a reductionist, monocellular C2C12 model, for *de novo* intrafusal skeletal muscle generation is presented. The addition of a recombinant, developmentally associated protein, Nrg-1, is used to replicate an innervating Ia afferent neuron. C2C12 myoblasts offer an accessible, fusion competent, reproducible cell line that are widely used in a number of skeletal muscle research applications. C2C12s facilitate rapid model progression, assay development and also an opportunity to investigate intrafusal fibre development in a reductionist pure myoblast population. Progress in such a cell line will facilitate translation to a primary human, multi-cell, integrated, biomimetic model of the muscle spindle.

To firstly investigate the utility of C2C12s as a model of intrafusal fibre development, Nrg-1 was added to developing C2C12 myotubes, which resulted in an over 4-fold increase of bag myotube formation (Figure 3C). This supports previous *in vivo* and *in vitro* experiments (Tourtellotte and Milbrandt, 1998; Tourtellotte et al., 2001; Jacobson et al., 2004; Albert et al., 2005; Rumsey et al., 2008; Akay et al., 2014; Herndon et al., 2014; Oliveira Fernandes and Tourtellotte, 2015; Colón et al., 2017, 2020; Guo et al., 2017) that indicate Nrg-1 is essential for the development of intrafusal skeletal muscle fibres. The fold increase in bag myotubes is comparable to Hickman and others, who reported 4-5-fold increases in both primary rat and human cells (Rumsey et al., 2008; Guo et al., 2017). However, the percentage of bag myotubes to the total myotube population is much higher in our C2C12 model compared to human myoblasts as previously published (41.61 ± 6.77% vs 15.80 ± 6.62%). Nevertheless, we must consider in this study, a novel statistical DDR method was used to more objectively define intrafusal bag myotubes, which may be a contributing factor, rather than suggesting C2C12s are more responsive to Nrg-1.

Myoblast fusion is a required event for skeletal muscle development and regeneration, therefore measuring fusion efficiency *in vitro* is a good indicator for overall myogenesis (Sampath et al., 2018). Fusion efficiency was not significantly changed upon Nrg-1 addition in this investigation (Figure 3C), which would suggest that the morphological changes observed are independent of other myogenic fusion parameters. There was however, a 1.4-fold increase in total nuclei number per FOV, which did not affect fusion efficiency (Figure 3B). This change can be accounted to the increase of bag myotubes, which contain on average over two more myonuclei than linear fibres (Figure 3C).

Nrg-1 downstream activation of Egr3 is a key signalling event in muscle spindle and intrafusal skeletal muscle fibre development (Tourtellotte and Milbrandt, 1998; Jacobson et al., 2004; Albert et al., 2005; Herndon et al., 2014; Oliveira Fernandes and Tourtellotte, 2015). Ablation of Erg-3 is accompanied by abnormal muscle spindles, reduced running performance and degradation of locomotor pattern in mice (Tourtellotte et al., 2001; Akay et al., 2014; Oliveira Fernandes and Tourtellotte, 2015). To further validate the role of Nrg-1 on C2C12 intrafusal bag myotube formation, Egr3 expression was quantified. Treated cells displayed a significant increase in gene (Figure 3D) and protein expression (Figure 6D) of Egr3, similar to that previously evidenced in primary human (Jacobson et al., 2004) and C2C12s (Williams and Jacobson, 2010; Herndon et al., 2014). Fluorescent micrographs demonstrated increased Egr3 protein staining intensity in Nrg-1 treated and intrafusal bag C2C12 myotubes, suggesting an important role for Egr3 toward C2C12 intrafusal bag myotube development. In contrast to the Hickman papers (Rumsey et al., 2008, 2010; Colón et al., 2017, 2020; Guo et al., 2017), control myotubes had visible expression of Egr3 (Figure 6A), meaning it was not a binary determinant of the intrafusal phenotype as defined by DDR. In previous studies, Egr3 displayed strong immunoreactivity in untreated myogenic cells (Herndon et al., 2014), is involved in *in vitro* myoblast proliferation (Kurosaka et al., 2017) and upregulated during differentiation (Muñoz et al., 2018; Choi et al., 2020). Therefore, non-binary staining results were expected. Egr3 in Figure 6, paired with the increase in intrafusal bag morphology (Figure 3B) suggests that Nrg-1 elicits endogenous production of Egr3, suggesting activation of the Nrg1/ErbB2/Egr3 signalling pathway, resulting in intrafusal specific differentiation.

To validate an intrafusal phenotype, the most in depth MyHC characterisation of Nrg-1 treated myotubes was completed. MyHCs are key contractile proteins and are major determinants of force velocity properties of muscle, which are differentially distributed across fibres and transiently expressed during development, regeneration and various stimulus such as injury or exercise (Schiaffino et al., 2015). Therefore, MyHCs are often used to provide an indication of the developing, regenerative or mature phenotype of skeletal muscle (Liu et al., 2005). *In vivo* studies have highlighted MyHC3, MyHC6, MyHC8 and MyHC7b/14 as having preferential or retained expression in mature intrafusal muscle fibres (Kucera et al., 1992; Liu et al., 2002; Österlund et al., 2011; Schiaffino and Reggiani, 2011; Thornell et al., 2015) and therefore are suitable candidates for characterisation *in vitro*. MyHC6 and MyHC8 have been formerly shown to display increased protein expression in human cells *in vitro* following Nrg-1 treatment (Jacobson et al., 2004), even in the absence of bag myotube morphology. *In vitro* bag myotubes have displayed MyHC6 specific staining compared to their morphologically linear neighbours in primary rat (Rumsey et al., 2008), primary human (Guo et al., 2017) and iPSCs (Colón et al., 2020). In contrast, this current paper presents that MyHC3,6 and 8 are all downregulated transcriptionally when analysed by RT-qPCR (Figures 4A,B), translationally when analysed by western blots (Figures 5A–C), and exhibit no clear preferential immunofluorescent staining in C2C12 myotubes with a bag morphology (Figures 5A–C). MyHC6 presented sparse staining in Nrg-1 vs control, as compared to the immature isoforms. Although MyHC6 is a predominately cardiac isoform, it is expressed in slow-twitch skeletal muscle tissue and its reduced expression in Nrg-1 treated cells could be related to a faster MyHC profile, as indicated by the increased Myh4 gene expression (Stuart et al., 2016) Furthermore, s46 (DSHB, United States), an antibody for avian slow developmental isoform, is repeatedly stated as being the best intrafusal specific marker *in vivo* and *in vitro* (Schiaffino and Reggiani, 2011; Schiaffino et al., 2015) gave no immunoreactivity in the present model (data not shown). The only Myh increase was a 2-fold increase in Myh4 following Nrg-1 treatment (Figure 4A), representing a rise from 33.24 ± 0.85% to 60.13 ± 0.82% of total Myh expression (Figure 4B). Myh4 codes for MyHC-IIb, the most prominent isoform (Pellegrino et al., 2003) expressed in adult mice, associated with high forces of contraction combined with rapid contractile characteristics (Harrison et al., 2011). MyHC4/IIb Immunofluorescence and western blotting were attempted using both BF-F3 (DSHB, United States) and 20140-1-AP (Proteintech, United Kingdom) antibodies. Unfortunately, they proved unsuccessful in achieving a clean and strong signal which would be sufficient for use. Intrafusal bag fibres, especially of the type 1 subset, are associated with a slow phenotype, however intrafusal chain and bag_2_ fibres express a fast phenotype in both human and rats (Kucera et al., 1992; Soukup et al., 1995; Liu et al., 2005; Österlund et al., 2013). Therefore, an increase in Myh4 is not indicative of poor intrafusal myotube generation in this model, but rather an indication of a more mature, glycolytic and faster contractile phenotype following Nrg-1 supplementation (Kucera et al., 1992; Soukup et al., 1995; Liu et al., 2005; Österlund et al., 2013). Furthermore, previous studies have demonstrated the increased expression of Myh4 mRNA in C2C12 myotubes in similar time course of experimentation (Brown et al., 2012). In addition, rat intrafusal fibres are believed to originate from three sequential generations of myotubes, the MyHC expression displays regional variability across the fibre length, differs throughout development and is dependent on interactions with external factors such as innervation and intracapsular niche (Kucera and Walro, 1990, 1995). This highlights the complexity of using MyHC expression to define an intrafusal myotube *in vitro* in the absence of sensory innervation and functional outputs (Kucera et al., 1992; Liu et al., 2002).

To characterise this further, supplementary intrafusal markers were employed. Myf5, an early myogenic regulatory factor, retains expression into adult intrafusal muscle fibres *in-vivo* (Zammit et al., 2004). Etv4 transcription factor, is another early stage intrafusal muscle fibre development marker, which similar to Egr3 is induced by afferent innervation (Arber et al., 2000; Hippenmeyer et al., 2002). However, Etv4 is largely involved in patterning motor innervation rather than in spindle morphogenesis *per se* (Albert et al., 2005). Gdnf is another established intrafusal fibre marker that has an essential role in fusimotor survival (Shneider et al., 2009; Schiaffino and Reggiani, 2011). Additionally, a murine knockdown model identified Sstr2 and Prph1 as novel intrafusal specific markers (Albert et al., 2005), which are yet to be utilised for *in vitro* characterisation. Despite this, gene expression changes for the aforementioned targets, aside from a significant 2-fold increase in Etv4 (Figure 6E), were insignificant following Nrg-1 treatment (Figures 3F, 6E). Activation of Etv4 expression further clarifies Nrg-1 is replicating afferent signalling pathways in this model. Although Myf5 gene expression was not modified, Myod1 was significantly upregulated. As an intermediate MRF, Myod1 is required for myogenic cells to exit the cell cycle and to enter the differentiation process (Zanou and Gailly, 2013). With the evidence for increased cell proliferation during the differentiation phase in Nrg-1 treated culture (Figure 7A), the increase in Myod1 compared to control could be accounted to a greater availability of myoblasts for fusion at the given time point. Myod1 also has a causative relationship with increased Myh4 expression in mice, which may also be a contributing factor to the upregulation of Myh4 discussed above (Wheeler et al., 1999; Seward et al., 2001; Ekmark et al., 2007; Zammit, 2017).

Intrafusal fibres have an increased expression of PAX 7 satellite cells and decreased myonuclear domain compared to their extrafusal neighbours (Kirkpatrick et al., 2008). This highlights the importance of satellite cell and myonuclei distribution to muscle spindle and intrafusal fibre function (Kirkpatrick et al., 2008). In corroboration to this hypothesis, adult mice ablated of satellite cells exhibit no detectable changes in extrafusal muscle fibre morphology, fibre-type composition, aerobic capacity or force generation. However, they display gross motor coordination defects, increased muscle spindle extracellular matrix deposition and decreased intrafusal fibre cross-sectional area (Jackson et al., 2015). A similar phenotype is seen with skeletal muscle specific Egr3 knockout mice, whom are deficient of a functional muscle spindle (Tourtellotte and Milbrandt, 1998; Akay et al., 2014; Oliveira Fernandes and Tourtellotte, 2015). Increased satellite cell number and smaller myonuclear domains are indicative of greater capacities for growth, regeneration, and repair (Rosser et al., 2002; Allouh et al., 2008; Shenkman et al., 2010). In general, higher satellite cells numbers corresponds with greater contractile activity (Gibson and Schultz, 1982, 1983; Yin et al., 2013) and myonuclear domains are smaller in slower, frequently activated fibres (Roy et al., 2005; Aravamudan et al., 2006). Therefore, intrafusal fibres, which are continuously sending and receiving information to the central nervous system, even at rest (Macefield and Knellwolf, 2018), fit these criteria. In this model, C2C12s express a significant upregulation in cell number (Figure 7A) and cell viability (Figure 7B) alongside reduced myotube area per myonuclei, following Nrg-1 treatment (Figure 7C). Therefore, recapitulating several *in vivo* phenotypes discussed above. Nrg-1/ErbB2 signalling activates many genes associated with specific cellular processes including proliferation, differentiation, survival, apoptosis, and migration (Geissler et al., 2020). To this extent, it has previously been associated with and not limited to, upregulating proliferation in myoblasts (Ford et al., 2003), fibroblasts (Kirabo et al., 2017), cardiomyocytes (Geissler et al., 2020), Schwann cells (Fallon et al., 2004) and epithelial cells (Liu and Kern, 2002). Additionally, Egr3 expression has been linked C2C12 myoblast proliferation (Kurosaka et al., 2017), therefore it is hard to speculate on the downstream signalling responsible for increased proliferation following Nrg-1 treatment, without further cell type specific investigations.

We must also consider the maturity of the model, during skeletal muscle development and regeneration, MyHCs and MRFs are transiently expressed, and results *in vitro* will vary depending on the culture environment and time spent differentiating before terminal analysis (Brown et al., 2012; Stuart et al., 2016; Rao et al., 2018). C2C12 myotubes after 8 days differentiation in both control and Nrg-1 treated conditions are still exhibiting an immature phenotype, as determined by high proportions of Myh3 gene expression (Figure 4B) and strong staining for MyHC3 and MyHC8 (Figures 5A,B). *In vivo* work used to identify intrafusal specific markers are mostly based from adult, or new-born immunohistochemistry samples (Kucera et al., 1992, 1993; Kucera and Walro, 1992, 1995; Tourtellotte and Milbrandt, 1998; Walro and Kucera, 1999; Liu et al., 2002; Zammit et al., 2004; Horst et al., 2006; Kirkpatrick et al., 2008, 2010; Oliveira Fernandes and Tourtellotte, 2015). To overcome this, future model iterations should look to increase the differentiation time span. 2D monolayer cultures will not facilitate this, as spontaneous contractions in C2C12 myotubes detach them from the surface when cultured much past 8 days. Therefore researchers should look to adopt a 3D tissue engineering approach, not only will this facilitate longer culture times (Vandenburgh et al., 1988; Juhas et al., 2014; Rao et al., 2018), but it will better replicate the *in vivo* environment, allowing cell to ECM and cell to cell interactions in three directions and give the user control over orientation, porosity and stiffness (Torii et al., 2018). In turn, 3D skeletal muscle cultures permit more mature molecular, structural and functional differentiation, better representing native adult muscle (Rao et al., 2018; Capel et al., 2019; Wang et al., 2019; Zhuang et al., 2020) and may facilitate identification of retained immature or specialised intrafusal specific phenotypes using C2C12 cells. Other novel future approaches are discussed in detail in a recent review paper by our group (Barrett et al., 2020).

Without a definitive intrafusal phenotype *in vitro*, we cannot speculate on the origins of intrafusal fibres. If a single bipotential population of myocytes can develop into both fibre type (i.e., intrafusal and extrafusal), then theoretically we should be able to achieve a pure, intrafusal-only C2C12 cell culture population. To achieve this, a reliable intrafusal linear and bag fibre marker is required. If a distinct lineage of myoblast intrinsically committed to differentiation into intrafusal fibres exists, then C2C12s may be an incompatible source for an intrafusal fibre model. The data attained in this study highlights several hallmarks of intrafusal fibres within Nrg-1 treated C2C12 myotubes. These warrant future endeavours using more sophisticated, biomimetic cell cultures to tissue engineer functional intrafusal muscle for the applications of both basic science and clinical studies, towards improving complete neuromuscular function in disease and injury.

## Conclusion

In conclusion, this study presents a novel minimalistic, monocellular C2C12 model for progression towards *de novo* intrafusal skeletal muscle generation. Recombinant addition of intrafusal muscle developmentally associated protein Nrg-1 was employed to replicate an innervating Ia afferent neuron. The exogenous addition of Nrg-1 elicits elevated endogenous production of Egr3, resulting in intrafusal-like specific differentiation, as determined by the novel morphological characterisation of intrafusal bag myotubes. Concurrently, Nrg-1 increased cell proliferation during the differentiation phase of the protocol, resulting in increased nuclei per FOV and paired with less myotube area per nuclei. Extensive mRNA and Protein analysis of MyHCs is strongly suggestive of a unique phenotype following Nrg-1 addition, however both control and treated myotubes remain in an immature state. All putative intrafusal specific targets apart from developmental proteins Egr3 and Etv4 are not preferentially expressed in Nrg-1 treated myotubes. Egr3 staining, although significantly increased, was not a binary determinant for intrafusal bag myotubes. The suitability for C2C12s to generate intrafusal muscle fibres is still unclear. There is enough promise from the results presented here to encourage future research toward biomimetic tissue engineering approaches, in the hope of producing mature, Nrg-1 treated myotubes, characteristic of native, *in vivo* intrafusal skeletal muscle. These future models could provide platforms for developmental or disease state studies, pre-clinical screening, or clinical applications, including regeneration or replacement of diseased or dysfunctional tissue (Barrett et al., 2020; Kröger and Watkins, 2021).

## Data Availability

The original contributions presented in the study are included in the, further inquiries can be directed to the corresponding author.

## References

[B1] Akay T. Tourtellotte W. G. Arber S. Jessell T. M. (2014). Degradation of Mouse Locomotor Pattern in the Absence of Proprioceptive Sensory Feedback. Proc. Natl. Acad. Sci. USA 111, 16877–16882. 10.1073/pnas.1419045111 25389309 PMC4250167

[B2] Albert Y. v. Whitehead J. Eldredge L. Carter J. Gao X. Tourtellotte W. G. (2005). Transcriptional Regulation of Myotube Fate Specification and Intrafusal Muscle Fiber Morphogenesis. J. Cel Biol. 169, 257–268. 10.1083/jcb.200501156 PMC2171871 15837802

[B3] Allouh M. Z. Yablonka-Reuveni Z. Rosser B. W. C. (2008). Pax7 Reveals a Greater Frequency and Concentration of Satellite Cells at the Ends of Growing Skeletal Muscle Fibers. J. Histochem. Cytochem. 56, 77–87. 10.1369/jhc.7A7301.2007 17938281 PMC2323121

[B4] Andrechek E. R. Hardy W. R. Girgis-Gabardo A. A. Perry R. L. S. Butler R. Graham F. L. (2002). ErbB2 Is Required for Muscle Spindle and Myoblast Cell Survival. Mol. Cel. Biol. 22, 4714–4722. 10.1128/MCB.22.13.4714-4722.2002 PMC133917 12052879

[B5] Aravamudan B. Mantilla C. B. Zhan W.-Z. Sieck G. C. (2006). Denervation Effects on Myonuclear Domain Size of Rat Diaphragm Fibers. J. Appl. Physiol. 100, 1617–1622. 10.1152/japplphysiol.01277.2005 16410375

[B6] Arber S. Ladle D. R. Lin J. H. Frank E. Jessell T. M. (2000). ETS Gene Er81 Controls the Formation of Functional Connections between Group Ia Sensory Afferents and Motor Neurons. Cell 101, 485–498. 10.1016/S0092-8674(00)80859-4 10850491

[B7] Banks R. W. (2015). The Innervation of the Muscle Spindle: a Personal History. J. Anat. 227, 115–135. 10.1111/joa.12297 26095428 PMC4523316

[B8] Banks R. W. (1994). The Motor Innervation of Mammalian Muscle Spindles. Prog. Neurobiol. 43, 323–362. 10.1016/0301-0082(94)90059-0 7816930

[B9] Barrett P. Quick T. J. Mudera V. Player D. J. (2020). Generating Intrafusal Skeletal Muscle Fibres *In Vitro*: Current State of the Art and Future Challenges. J. Tissue Eng. 11, 204173142098520. 10.1177/2041731420985205 PMC8693220 34956586

[B10] Bland J. M. Altman D. G. (1996). Statistics Notes: Transforming Data. BMJ 312, 770. 10.1136/bmj.312.7033.770 8605469 PMC2350481

[B11] Brown D. M. Parr T. Brameld J. M. (2012). Myosin Heavy Chain mRNA Isoforms Are Expressed in Two Distinct Cohorts during C2C12 Myogenesis. J. Muscle Res. Cel Motil. 32, 383–390. 10.1007/s10974-011-9267-4 22012579

[B12] Bullinger K. L. Nardelli P. Pinter M. J. Alvarez F. J. Cope T. C. (2011). Permanent central Synaptic Disconnection of Proprioceptors after Nerve Injury and Regeneration. II. Loss of Functional Connectivity with Motoneurons. J. Neurophysiol. 106, 2471–2485. 10.1152/jn.01097.2010 21832030 PMC3214087

[B13] Bustin S. A. Benes V. Garson J. A. Hellemans J. Huggett J. Kubista M. (2009). The MIQE Guidelines: Minimum Information for Publication of Quantitative Real-Time PCR Experiments. Clin. Chem. 55, 611–622. 10.1373/clinchem.2008.112797 19246619

[B14] Capel A. J. Rimington R. P. Fleming J. W. Player D. J. Baker L. A. Turner M. C. (2019). Scalable 3D Printed Molds for Human Tissue Engineered Skeletal Muscle. Front. Bioeng. Biotechnol. 7, 20. 10.3389/fbioe.2019.00020 30838203 PMC6383409

[B15] Chal J. Pourquié O. (2017). Making Muscle: Skeletal Myogenesis *In Vivo* and *In Vitro* . Development 144, 2104–2122. 10.1242/DEV.151035 28634270

[B16] Chen H.-H. Tourtellotte W. G. Frank E. (2002). Muscle Spindle-Derived Neurotrophin 3 Regulates Synaptic Connectivity between Muscle Sensory and Motor Neurons. J. Neurosci. 22, 3512–3519. 10.1523/JNEUROSCI.22-09-03512.2002 11978828 PMC6758400

[B17] Choi I. Y. Lim H. Cho H. J. Oh Y. Chou B.-K. Bai H. (2020). Transcriptional Landscape of Myogenesis from Human Pluripotent Stem Cells Reveals a Key Role of TWIST1 in Maintenance of Skeletal Muscle Progenitors. Elife 9. 10.7554/eLife.46981 PMC6996923 32011235

[B18] Colón A. Badu-Mensah A. Guo X. Goswami A. Hickman J. J. (2020). Differentiation of Intrafusal Fibers from Human Induced Pluripotent Stem Cells. ACS Chem. Neurosci. 11, 1085–1092. 10.1021/acschemneuro.0c00055 32159941

[B19] Colón A. Guo X. Akanda N. Cai Y. Hickman J. J. (2017). Functional Analysis of Human Intrafusal Fiber Innervation by Human γ-motoneurons. Sci. Rep. 7, 17202. 10.1038/s41598-017-17382-2 29222416 PMC5722897

[B20] Conte A. Khan N. Defazio G. Rothwell J. C. Berardelli A. (2013). Pathophysiology of Somatosensory Abnormalities in Parkinson Disease. Nat. Rev. Neurol. 9, 687–697. 10.1038/nrneurol.2013.224 24217516

[B21] Cope T. C. Bonasera S. J. Nichols T. R. (1994). Reinnervated Muscles Fail to Produce Stretch Reflexes. J. Neurophysiol. 71, 817–820. 10.1152/jn.1994.71.2.817 8176445

[B22] D'Silva L. J. Lin J. Staecker H. Whitney S. L. Kluding P. M. (2016). Impact of Diabetic Complications on Balance and Falls: Contribution of the Vestibular System. Phys. Ther. 96, 400–409. 10.2522/ptj.20140604 26251477 PMC4774386

[B23] Ekmark M. Rana Z. A. Stewart G. Hardie D. G. Gundersen K. (2007). De-phosphorylation of MyoD Is Linking Nerve-Evoked Activity to Fast Myosin Heavy Chain Expression in Rodent Adult Skeletal Muscle. J. Physiol. 584, 637–650. 10.1113/jphysiol.2007.141457 17761773 PMC2277165

[B24] Ettinger L. R. Boucher A. Simonovich E. (2018). Patients with Type 2 Diabetes Demonstrate Proprioceptive Deficit in the Knee. Wjd 9, 59–65. 10.4239/wjd.v9.i3.59 29607003 PMC5876505

[B25] Fallon K. B. Havlioglu N. Hamilton L. H. Cheng T. P. H. Carroll S. L. (2004). Constitutive Activation of the neuregulin-1/erbB Signaling Pathway Promotes the Proliferation of a Human Peripheral Neuroepithelioma Cell Line. J. Neurooncol. 66, 273–284. 10.1023/b:neon.0000014521.28294.84 15015657

[B26] Ferlinc A. Fabiani E. Velnar T. Gradisnik L. (2019). The Importance and Role of Proprioception in the Elderly: a Short Review. Mater. Sociomed 31, 219. 10.5455/msm.2019.31.219-221 31762707 PMC6853739

[B27] Feys P. Helsen W. Ilsbroukx S. Meurrens T. (2011). Is MS Intention Tremor Amplitude Related to Changed Peripheral Reflexes? ISRN Neurol. 2011, 1–7. 10.5402/2011/192414 PMC3263540 22389808

[B28] Fling B. W. Dutta G. G. Schlueter H. Cameron M. H. Horak F. B. (2014). Associations between Proprioceptive Neural Pathway Structural Connectivity and Balance in People with Multiple Sclerosis. Front. Hum. Neurosci. 8, 1–11. 10.3389/fnhum.2014.00814 25368564 PMC4202774

[B29] Ford B. D. Han B. Fischbach G. D. (2003). Differentiation-dependent Regulation of Skeletal Myogenesis by Neuregulin-1. Biochem. Biophysical Res. Commun. 306, 276–281. 10.1016/S0006-291X(03)00964-1 12788100

[B30] Geissler A. Ryzhov S. Sawyer D. B. (2020). Neuregulins: Protective and Reparative Growth Factors in Multiple Forms of Cardiovascular Disease. Clin. Sci. 134, 2623–2643. 10.1042/CS20200230 PMC7557502 33063822

[B31] Gibson M. C. Schultz E. (1983). Age-related Differences in Absolute Numbers of Skeletal Muscle Satellite Cells. Muscle Nerve 6, 574–580. 10.1002/mus.880060807 6646160

[B32] Gibson M. C. Schultz E. (1982). The Distribution of Satellite Cells and Their Relationship to Specific Fiber Types in Soleus and Extensor Digitorum Longus Muscles. Anat. Rec. 202, 329–337. 10.1002/ar.1092020305 7072981

[B33] Guo X. Colon A. Akanda N. Spradling S. Stancescu M. Martin C. (2017). Tissue Engineering the Mechanosensory Circuit of the Stretch Reflex Arc with Human Stem Cells: Sensory Neuron Innervation of Intrafusal Muscle Fibers. Biomaterials 122, 179–187. 10.1016/j.biomaterials.2017.01.005 28129596 PMC5299592

[B34] Haftel V. K. (2005). Central Suppression of Regenerated Proprioceptive Afferents. J. Neurosci. 25, 4733–4742. 10.1523/JNEUROSCI.4895-04.2005 15888649 PMC6724774

[B35] Hardin J. W. Hilbe J. M. (2014). Regression Models for Count Data Based on the Negative Binomial(p) Distribution. Stata J. 14, 280–291. 10.1177/1536867X1401400203

[B36] Harrison B. C. Allen D. L. Leinwand L. A. (2011). IIb or Not IIb? Regulation of Myosin Heavy Chain Gene Expression in Mice and Men. Skelet. Muscle 1, 5–9. 10.1186/2044-5040-1-5/FIGURES/3 21798083 PMC3143903

[B37] Herndon C. A. Ankenbruck N. Fromm L. (2014). The Erk MAP Kinase Pathway Is Activated at Muscle Spindles and Is Required for Induction of the Muscle Spindle‐specific Gene Egr3 by Neuregulin1. J. Neurosci. Res. 92, 174–184. 10.1002/jnr.23293 24272970

[B38] Hildyard J. C. Wells D. J. (2014). Identification and Validation of Quantitative PCR Reference Genes Suitable for Normalizing Expression in normal and Dystrophic Cell Culture Models of Myogenesis. Plos Curr. 6, faafdde4bea8df4aa7d06cd5553119a6. 10.1371/currents.md.faafdde4bea8df4aa7d06cd5553119a6 PMC3948689 24634799

[B39] Hippenmeyer S. Shneider N. A. Birchmeier C. Burden S. J. Jessell T. M. Arber S. (2002). A Role for Neuregulin1 Signaling in Muscle Spindle Differentiation. Neuron 36, 1035–1049. 10.1016/S0896-6273(02)01101-7 12495620

[B40] Horst D. Ustanina S. Sergi C. Mikuz G. Juergens H. Braun T. (2006). Comparative Expression Analysis of Pax3 and Pax7 during Mouse Myogenesis. Int. J. Dev. Biol. 50, 47–54. 10.1387/ijdb.052111dh 16323077

[B41] Jackson J. R. Kirby T. J. Fry C. S. Cooper R. L. McCarthy J. J. Peterson C. A. (2015). Reduced Voluntary Running Performance Is Associated with Impaired Coordination as a Result of Muscle Satellite Cell Depletion in Adult Mice. Skeletal Muscle 5, 41. 10.1186/s13395-015-0065-3 26579218 PMC4647638

[B42] Jacobson C. Duggan D. Fischbach G. (2004). Neuregulin Induces the Expression of Transcription Factors and Myosin Heavy Chains Typical of Muscle Spindles in Cultured Human Muscle. Proc. Natl. Acad. Sci. 101, 12218–12223. 10.1073/pnas.0404240101 15302938 PMC514402

[B43] Juhas M. Engelmayr G. C. Fontanella A. N. Palmer G. M. Bursac N. (2014). Biomimetic Engineered Muscle with Capacity for Vascular Integration and Functional Maturation *In Vivo* . Proc. Natl. Acad. Sci. 111, 5508–5513. 10.1073/pnas.1402723111 24706792 PMC3992675

[B44] Kirabo A. Ryzhov S. Gupte M. Sengsayadeth S. Gumina R. J. Sawyer D. B. (2017). Neuregulin-1β Induces Proliferation, Survival and Paracrine Signaling in normal Human Cardiac Ventricular Fibroblasts. J. Mol. Cell Cardiol. 105, 59–69. 10.1016/J.YJMCC.2017.03.001 28263756 PMC5715731

[B45] Kirkpatrick L. J. Allouh M. Z. Nightingale C. N. Devon H. G. Yablonka-Reuveni Z. Rosser B. W. C. (2008). Pax7 Shows Higher Satellite Cell Frequencies and Concentrations within Intrafusal Fibers of Muscle Spindles. J. Histochem. Cytochem. 56, 831–840. 10.1369/jhc.2008.951608 18541708 PMC2516954

[B46] Kirkpatrick L. J. Yablonka-Reuveni Z. Rosser B. W. C. (2010). Retention of Pax3 Expression in Satellite Cells of Muscle Spindles. J. Histochem. Cytochem. 58, 317–327. 10.1369/jhc.2009.954792 20026670 PMC2842595

[B47] Kozeka K. Ontell M. (1981). The Three-Dimensional Cytoarchitecture of Developing Murine Muscle Spindles. Developmental Biol. 87, 133–147. 10.1016/0012-1606(81)90067-1 7286415

[B48] Kröger S. Watkins B. (2021). Muscle Spindle Function in Healthy and Diseased Muscle. Skeletal Muscle 11, 3. 10.1186/s13395-020-00258-x 33407830 PMC7788844

[B49] Kucera J. Walro J. M. (1992). Formation of Muscle Spindles in the Absence of Motor Innervation. Neurosci. Lett. 145, 47–50. 10.1016/0304-3940(92)90200-Q 1461566

[B50] Kucera J. Walro J. M. Gorza L. (1992). Expression of Type-specific MHC Isoforms in Rat Intrafusal Muscle Fibers. J. Histochem. Cytochem. 40, 293–307. 10.1177/40.2.1552171 1552171

[B51] Kucera J. Walro J. M. (1990). Origin of Intrafusal Muscle Fibers in the Rat. Histochemistry 93, 567–580. 10.1007/BF00272199 2329055

[B52] Kucera J. Walro J. (1995). Origin of Intrafusal Fibers from a Subset of Primary Myotubes in the Rat. Anat. Embryol. 192, 149–158. 10.1007/BF00186003 7486011

[B53] Kucera J. Walro J. Reichler J. (1993). Differential Effects of Neonatal Denervation on Intrafusal Muscle Fibers in the Rat. Anat. Embryol. 187, 397–408. 10.1007/BF00185898 8512092

[B54] Kurosaka M. Ogura Y. Funabashi T. Akema T. (2017). Early Growth Response 3 (Egr3) Contributes a Maintenance of C2C12 Myoblast Proliferation. J. Cel. Physiol. 232, 1114–1122. 10.1002/jcp.25574 27576048

[B55] Leu M. Bellmunt E. Schwander M. Fariñas I. Brenner H. R. Müller U. (2003). Erbb2 Regulates Neuromuscular Synapse Formation and Is Essential for Muscle Spindle Development. Development 130, 2291–2301. 10.1242/dev.00447 12702645

[B56] Liu J.-X. Eriksson P.-O. Thornell L.-E. Pedrosa-Domellöf F. (2005). Fiber Content and Myosin Heavy Chain Composition of Muscle Spindles in Aged Human Biceps Brachii. J. Histochem. Cytochem. 53, 445–454. 10.1369/jhc.4A6257.2005 15805419

[B57] Liu J.-X. Eriksson P.-O. Thornell L.-E. Pedrosa-Domellöf F. (2002). Myosin Heavy Chain Composition of Muscle Spindles in Human Biceps Brachii. J. Histochem. Cytochem. 50, 171–183. 10.1177/002215540205000205 11799136

[B58] Liu J. Kern J. A. (2002). Neuregulin-1 Activates the JAK-STAT Pathway and Regulates Lung Epithelial Cell Proliferation. Am. J. Respir. Cel Mol. Biol. 27, 306–313. 10.1165/rcmb.4850 12204892

[B59] Maas H. Prilutsky B. I. Nichols T. R. Gregor R. J. (2007). The Effects of Self-Reinnervation of Cat Medial and Lateral Gastrocnemius Muscles on Hindlimb Kinematics in Slope Walking. Exp. Brain Res. 181, 377–393. 10.1007/s00221-007-0938-8 17406860 PMC2712217

[B60] Macefield V. G. Knellwolf T. P. (2018). Functional Properties of Human Muscle Spindles. J. Neurophysiol. 120, 452–467. 10.1152/jn.00071.2018.-Muscle 29668385

[B61] Miwa T. Miwa Y. Kanda K. (1995). Dynamic and Static Sensitivities of Muscle Spindle Primary Endings in Aged Rats to Ramp Stretch. Neurosci. Lett. 201, 179–182. 10.1016/0304-3940(95)12165-X 8848247

[B62] Muller K. A. Ryals J. M. Feldman E. L. Wright D. E. (2008). Abnormal Muscle Spindle Innervation and Large-Fiber Neuropathy in Diabetic Mice. Diabetes 57, 1693–1701. 10.2337/db08-0022 18362211

[B63] Muñoz M. García-Casco J. M. Caraballo C. Fernández-Barroso M. Á. Sánchez-Esquiliche F. Gómez F. (2018). Identification of Candidate Genes and Regulatory Factors Underlying Intramuscular Fat Content through Longissimus Dorsi Transcriptome Analyses in Heavy Iberian Pigs. Front. Genet. 9, 608. 10.3389/fgene.2018.00608 30564273 PMC6288315

[B64] Muramatsu K. Niwa M. Tamaki T. Ikutomo M. Masu Y. Hasegawa T. (2017). Effect of Streptozotocin-Induced Diabetes on Motoneurons and Muscle Spindles in Rats. Neurosci. Res. 115, 21–28. 10.1016/j.neures.2016.10.004 27826051

[B65] Oliveira Fernandes M. Tourtellotte W. G. (2015). Egr3-dependent Muscle Spindle Stretch Receptor Intrafusal Muscle Fiber Differentiation and Fusimotor Innervation Homeostasis. J. Neurosci. 35, 5566–5578. 10.1523/JNEUROSCI.0241-15.2015 25855173 PMC4388921

[B66] Österlund C. Liu J.-X. Thornell L.-E. Eriksson P.-O. (2013). Intrafusal Myosin Heavy Chain Expression of Human Masseter and Biceps Muscles at Young Age Shows Fundamental Similarities but Also Marked Differences. Histochem. Cel Biol. 139, 895–907. 10.1007/s00418-012-1072-7 23306907

[B67] Österlund C. Liu J.-X. Thornell L.-E. Eriksson P.-O. (2011). Muscle Spindle Composition and Distribution in Human Young Masseter and Biceps Brachii Muscles Reveal Early Growth and Maturation. Anat. Rec. 294, 683–693. 10.1002/ar.21347 21370492

[B68] Ovalle W. K. Dow P. R. (1986). Alterations in Muscle Spindle Morphology in Advanced Stages of Murine Muscular Dystrophy. Anat. Rec. 216, 111–126. 10.1002/ar.1092160202 2946251

[B69] Pellegrino M. A. Canepari M. Rossi R. D'Antona G. Reggiani C. Bottinelli R. (2003). Orthologous Myosin Isoforms and Scaling of Shortening Velocity with Body Size in Mouse, Rat, Rabbit and Human Muscles. J. Physiol. 546, 677–689. 10.1113/jphysiol.2002.027375 12562996 PMC2342590

[B70] Prather J. F. Nardelli P. Nakanishi S. T. Ross K. T. Nichols T. R. Pinter M. J. (2011). Recovery of Proprioceptive Feedback from Nerve Crush. J. Physiol. 589, 4935–4947. 10.1113/jphysiol.2011.210518 21788349 PMC3224884

[B71] Qiao Y. Cong M. Li J. Li H. Li Z. (2018). The Effects of Neuregulin-1β on Intrafusal Muscle Fiber Formation in Neuromuscular Coculture of Dorsal Root Ganglion Explants and Skeletal Muscle Cells. Skeletal Muscle 8, 29. 10.1186/s13395-018-0175-9 30219099 PMC6139134

[B72] Rao L. Qian Y. Khodabukus A. Ribar T. Bursac N. (2018). Engineering Human Pluripotent Stem Cells into a Functional Skeletal Muscle Tissue. Nat. Commun. 9, 1–12. 10.1038/s41467-017-02636-4 29317646 PMC5760720

[B73] Rio D. C. Ares M. Hannon G. J. Nilsen T. W. (2010). Purification of RNA Using TRIzol (TRI Reagent). Cold Spring Harb. Protoc. 2010, pdb.prot5439. 10.1101/pdb.prot5439 20516177

[B74] Rosser B. W. Dean M. S. Bandman E. (2002). Myonuclear Domain Size Varies along the Lengths of Maturing Skeletal Muscle Fibers. Int. J. Dev. Biol. 46, 747–754. 10.1387/IJDB.12216987 12216987

[B75] Roy R. R. Zhong H. Siengthai B. Edgerton V. R. (2005). Activity-dependent Influences Are Greater for Fibers in Rat Medial Gastrocnemius Than Tibialis Anterior Muscle. Muscle Nerve 32, 473–482. 10.1002/mus.20369 15962333

[B76] Ruijs A. C. J. Jaquet J.-B. Kalmijn S. Giele H. Hovius S. E. R. (2005). Median and Ulnar Nerve Injuries: A Meta-Analysis of Predictors of Motor and Sensory Recovery after Modern Microsurgical Nerve Repair. Plast. Reconstr. Surg. 116, 484–494. 10.1097/01.prs.0000172896.86594.07 16079678

[B77] Rumsey J. W. Das M. Bhalkikar A. Stancescu M. Hickman J. J. (2010). Tissue Engineering the Mechanosensory Circuit of the Stretch Reflex Arc: Sensory Neuron Innervation of Intrafusal Muscle Fibers. Biomaterials 31, 8218–8227. 10.1016/j.biomaterials.2010.07.027 20708792 PMC2941211

[B78] Rumsey J. W. Das M. Kang J.-F. Wagner R. Molnar P. Hickman J. J. (2008). Tissue Engineering Intrafusal Fibers: Dose- and Time-dependent Differentiation of Nuclear Bag Fibers in a Defined *In Vitro* System Using Neuregulin 1-β-1. Biomaterials 29, 994–1004. 10.1016/j.biomaterials.2007.10.042 18076984 PMC2276654

[B79] Sampath S. C. Sampath S. C. Millay D. P. (2018). Myoblast Fusion Confusion: The Resolution Begins. Skeletal Muscle 8, 3. 10.1186/s13395-017-0149-3 29386054 PMC5793351

[B80] Schiaffino S. Reggiani C. (2011). Fiber Types in Mammalian Skeletal Muscles. Physiol. Rev. 91, 1447–1531. 10.1152/physrev.00031.2010 22013216

[B81] Schiaffino S. Rossi A. C. Smerdu V. Leinwand L. A. Reggiani C. (2015). Developmental Myosins: Expression Patterns and Functional Significance. Skeletal Muscle 5, 22. 10.1186/s13395-015-0046-6 26180627 PMC4502549

[B82] Schindelin J. Arganda-Carreras I. Frise E. Kaynig V. Longair M. Pietzsch T. (2012). Fiji: An Open-Source Platform for Biological-Image Analysis. Nat. Methods 9, 676–682. 10.1038/nmeth.2019 22743772 PMC3855844

[B83] Schmittgen T. D. Livak K. J. (2008). Analyzing Real-Time PCR Data by the Comparative CT Method. Nat. Protoc. 3, 1101–1108. 10.1038/nprot.2008.73 18546601

[B84] Seward D. J. Haney J. C. Rudnicki M. A. Swoap S. J. (2001). bHLH Transcription Factor MyoD Affects Myosin Heavy Chain Expression Pattern in a Muscle-specific Fashion. Am. J. Physiology-Cell Physiol. 280, C408–C413. 10.1152/ajpcell.2001.280.2.c408 11208536

[B85] Shaffer S. W. Harrison A. L. (2007). Aging of the Somatosensory System: A Translational Perspective. Phys. Ther. 87, 193–207. 10.2522/ptj.20060083 17244695

[B86] Shenkman B. S. Turtikova O. V. Nemirovskaya T. L. Grigoriev A. I. (2010). Skeletal Muscle Activity and the Fate of Myonuclei. Acta Naturae 2, 59–65. 10.32607/20758251-2010-2-2-59-65 22649641 PMC3347558

[B87] Shneider N. A. Brown M. N. Smith C. A. Pickel J. Alvarez F. J. (2009). Gamma Motor Neurons Express Distinct Genetic Markers at Birth and Require Muscle Spindle-Derived GDNF for Postnatal Survival. Neural Dev. 4, 42. 10.1186/1749-8104-4-42 19954518 PMC2800842

[B88] Soukup T. Pedrosa-Domellöf F. Thornell L.-E. (1995). Expression of Myosin Heavy Chain Isoforms and Myogenesis of Intrafusal Fibres in Rat Muscle Spindles. Microsc. Res. Tech. 30, 390–407. 10.1002/jemt.1070300506 7787238

[B89] Stuart C. A. Stone W. L. Howell M. E. A. Brannon M. F. Hall H. K. Gibson A. L. (2016). Myosin Content of Individual Human Muscle Fibers Isolated by Laser Capture Microdissection. Am. J. Physiology-Cell Physiol. 310, C381–C389. 10.1152/ajpcell.00317.2015 PMC4971827 26676053

[B90] Taylor S. C. Berkelman T. Yadav G. Hammond M. (2013). A Defined Methodology for Reliable Quantification of Western Blot Data. Mol. Biotechnol. 55, 217–226. 10.1007/s12033-013-9672-6 23709336 PMC3840294

[B91] Teasdale H. Preston E. Waddington G. (2017). Proprioception of the Ankle Is Impaired in People with Parkinson's Disease. Mov. Disord. Clin. Pract. 4, 524–528. 10.1002/mdc3.12464 30363454 PMC6174440

[B92] Teixeira C. E. Duarte J. A. (2011). Myonuclear Domain in Skeletal Muscle Fibers. A Critical Review. Aehd 2, 92–101. 10.5628/aehd.v2i2.24

[B93] Thornell L. E. Carlsson L. Eriksson P. O. Liu J. X. Österlund C. Stål P. (2015). Fibre Typing of Intrafusal Fibres. J. Anat. 227, 136–156. 10.1111/joa.12338 26179023 PMC4523317

[B94] Torii R. Velliou R.-I. Hodgson D. Mudera V. (2018). Modelling Multi-Scale Cell-Tissue Interaction of Tissue-Engineered Muscle Constructs. J. Tissue Eng. 9, 204173141878714. 10.1177/2041731418787141 PMC6090492 30128109

[B95] Tourtellotte W. G. Keller-Peck C. Milbrandt J. Kucera J. (2001). The Transcription Factor Egr3 Modulates Sensory Axon-Myotube Interactions during Muscle Spindle Morphogenesis. Developmental Biol. 232, 388–399. 10.1006/dbio.2001.0202 11401400

[B96] Tourtellotte W. G. Milbrandt J. (1998). Sensory Ataxia and Muscle Spindle Agenesis in Mice Lacking the Transcription Factor Egr3. Nat. Genet. 20, 87–91. 10.1038/1757 9731539

[B97] van Deursen R. W. M. Sanchez M. M. Ulbrecht J. S. Cavanagh P. R. (1998). The Role of Muscle Spindles in Ankle Movement Perception in Human Subjects with Diabetic Neuropathy. Exp. Brain Res. 120, 1–8. 10.1007/s002210050371 9628397

[B98] van Deursen R. W. M. Simoneau G. G. (1999). Foot and Ankle Sensory Neuropathy, Proprioception, and Postural Stability. J. Orthop. Sports Phys. Ther. 29, 718–726. 10.2519/jospt.1999.29.12.718 10612069

[B99] Vandenburgh H. H. Karlisch P. Farr L. (1988). Maintenance of Highly Contractile Tissue-Cultured Avian Skeletal Myotubes in Collagen Gel. *In Vitro* Cel Dev Biol 24, 166–174. 10.1007/BF02623542 3350785

[B100] Walro J. M. Kucera J. (1999). Why Adult Mammalian Intrafusal and Extrafusal Fibers Contain Different Myosin Heavy-Chain Isoforms. Trends Neurosciences 22, 180–184. 10.1016/s0166-2236(98)01339-3 10203856

[B101] Wang J. Khodabukus A. Rao L. Vandusen K. Abutaleb N. Bursac N. (2019). Engineered Skeletal Muscles for Disease Modeling and Drug Discovery. Biomaterials 221, 119416. 10.1016/j.biomaterials.2019.119416 31419653 PMC7041662

[B102] Wheeler M. T. Snyder E. C. Patterson M. N. Swoap S. J. (1999). An E-Box within the MHC IIB Gene Is Bound by MyoD and Is Required for Gene Expression in Fast Muscle. Am. J. Physiology-Cell Physiol. 276, C1069–C1078. 10.1152/ajpcell.1999.276.5.c1069 10329954

[B103] Williams S. Jacobson C. (2010). α-Dystroglycan Is Essential for the Induction of Egr3, a Transcription Factor Important in Muscle Spindle Formation. Devel Neurobio 70, NA. 10.1002/dneu.20793 20213761

[B104] Ye J. Coulouris G. Zaretskaya I. Cutcutache I. Rozen S. Madden T. L. (2012). Primer-BLAST: a Tool to Design Target-specific Primers for Polymerase Chain Reaction. BMC Bioinformatics 13, 134. 10.1186/1471-2105-13-134 22708584 PMC3412702

[B105] Yin H. Price F. Rudnicki M. A. (2013). Satellite Cells and the Muscle Stem Cell Niche. Physiol. Rev. 93, 23–67. 10.1152/physrev.00043.2011 23303905 PMC4073943

[B106] Zammit P. S. Carvajal J. J. Golding J. P. Morgan J. E. Summerbell D. Zolnerciks J. (2004). Myf5 Expression in Satellite Cells and Spindles in Adult Muscle Is Controlled by Separate Genetic Elements. Developmental Biol. 273, 454–465. 10.1016/j.ydbio.2004.05.038 15328025

[B107] Zammit P. S. (2017). Function of the Myogenic Regulatory Factors Myf5, MyoD, Myogenin and MRF4 in Skeletal Muscle, Satellite Cells and Regenerative Myogenesis. Semin. Cel Developmental Biol. 72, 19–32. 10.1016/j.semcdb.2017.11.011 29127046

[B108] Zanou N. Gailly P. (2013). Skeletal Muscle Hypertrophy and Regeneration: Interplay between the Myogenic Regulatory Factors (MRFs) and Insulin-like Growth Factors (IGFs) Pathways. Cell. Mol. Life Sci. 70, 4117–4130. 10.1007/s00018-013-1330-4 23552962 PMC11113627

[B109] Zhou Y. Liu D. Kaminski H. J. (2010). Myosin Heavy Chain Expression in Mouse Extraocular Muscle: More Complex Than Expected. Invest. Ophthalmol. Vis. Sci. 51, 6355–6363. 10.1167/iovs.10-5937 20610840 PMC3055759

[B110] Zhuang P. An J. Chua C. K. Tan L. P. (2020). Bioprinting of 3D *In Vitro* Skeletal Muscle Models: A Review. Mater. Des. 193, 108794. 10.1016/j.matdes.2020.108794

